# Supplementary material for: Monometallic interphasic synergy via nano-hetero-interfacing for hydrogen evolution in alkaline electrolytes
Source: Nat Commun. 2023 Feb 1;14:547. doi: 10.1038/s41467-023-36100-3 (PMC9892594; doi:10.1038/s41467-023-36100-3)
Supplement: Supplementary file 1 — Supplementary Information [file 41467_2023_36100_MOESM1_ESM.pdf]

Supporting Information

for

Monometallic interphasic synergy via nano-hetero-interfacing for alkaline hydrogen evolution reaction

Kamran Dastafkan,[1] Xiangjian Shen,[2] Rosalie K. Hocking,[3] Quentin Meyer,[1] Chuan Zhao[1,*]

Email: chuan.zhao@unsw.edu.au

Supplementary Methods

Preparation of $Ni(OH)_2$@Ni-N/Ni-C/Ni/CFP and $Ni(OH)_2$/Ni/CFP

To synthesize Ni nanostructures on inert substrates, an ultrathin metallic Ni layer was electrodeposited on carbon fiber paper (CFP, 1x3 cm^2) in a 50 mL aqueous solution containing 0.5 M $NiSO_4$ and 0.5 M H_3BO_3 using CHI 760D electrochemical workstation 2.0 V (vs. SCE) for 600 s at room temperature. Afterwards, the aforementioned solvothermal synthesis and post-thermal annealing were performed on Ni/CFP substrates to acquire $Ni(OH)_2$@Ni-N/Ni-C/Ni/CFP and $Ni(OH)_2$/Ni/CFP electrodes.

Preparation of Pt/C/NF

For comparison, 5 mg commercial Pt/C powder was dispersed in the mixture of 475 μL DI water, 500 μL ethanol, and 25 μL 5 wt.% Nafion as binder to form a uniform ink. Then, 20 wt.% Pt/C/NF electrode was prepared by drop-casting the ink onto NF (0.25 cm^2).

Material characterizations

X-ray diffraction (XRD) patterns were obtained in thin film mode (Grazing incidence, GIXRD) using an Empyrean PANalytical diffractometer (CuKα radiation, 45 kV, 40 mA, $\lambda = 1.540598$ Å). Scanning electron microscopy (SEM) and energy dispersive X-ray spectrometry (EDS) were performed using a Joel 7001 microscope. Pseudo-coloring is used for the SEM images. Focused ion beam-scanning transmission electron microscopy (FIB-STEM), transmission electron microscopy (TEM) with high resolution (HRTEM) and selected area electron diffraction (SAED), as well as high-angle annular dark-field scanning transmission electron microscopy-energy dispersive X-ray spectrometry for elemental mapping (HAADF-STEM-EDS) were conducted by a JEOL JEM-F200 Multi-Purpose FEG-S/TEM operating at an accelerating voltage of 200 kV. X-

ray photoelectron spectroscopy (XPS) and ultraviolet photoelectron spectroscopy (UPS) were carried out using an ESCALAB 250 Xi, Thermo Scientific spectrometer. The passing energy used for XPS was 100 eV for survey scans or 20 eV for region scans, and 2eV for UPS. X-ray source was mono-chromated Al K alpha (energy 1486.68 eV) and ultraviolet source was He I (energy 21.2eV) and He II (energy 40.8eV). Raman spectroscopy was performed using a Renishaw inVia spectrometer using a 514 nm laser.

Experimental design modelling

Design of experiment (DoE) is performed to optimize the nano-hetero-interfacing. Response surface methodology (RSM) is conducted using Taguchi plan.[1,2] The influence of four control parameters during solvothermal synthesis is investigated with three observation levels (low, medium, and high) in DoE optimization (**Table S1**). Considering the highest degree of interaction, a fully balanced factorial design is implemented to assign three levels for each control parameter. In this model, the number of permutations is 8, as in the degrees of freedom is 9-1 = 8. Normalized variables (X_1, X_2, X_3 and X_4) are introduced to represent each control parameter with low, medium, and high levels equated as -1, 0 and 1 (**Table S2** and **Table S3**). Using the DoE model, the linear (first order) and quadratic (second order) influence of each control parameter on the input energy (overpotential) are investigated at a certain current response.

$$V(X_1, X_2, X_3, X_4) = A_0 + A_1X_1 + B_1X_2 + C_1X_3 + D_1X_4 + A_2X_1^2 + B_2X_2^2 + C_2X_3^2 + D_2X_4^2 \quad \text{(S1)}$$

where A_0 is the measured overpotential for the influence of all control parameters at medium level. A_1 to D_1 are the weighted coefficients of the linear interactions, and A_2 to D_2 are the weighted coefficients of the quadratic interactions. Thirty-five additional experiments were carried out

within the domain described in **Table S1**. The measured overpotentials for the total 44 experiments performed in the DoE model were optimized at a current density of -100 mA cm^{-2}.

Electrochemically active surface area (ECSA)

The active site density of the as-prepared samples was assessed by their ECSA by measuring the corresponding vales of double-layer capacitance (C_{DL}) in 1.0 M KOH. Cyclic voltammograms were recorded at non-faradaic potential ranges with increasing scan rate from 10 to 180 mV s^{-1}. The slope of linear regressions of cathodic current responses (i_c) as a function of scan rate (ʋ) determines the corresponding C_{DL} values according to the following equation:

$$i_a = \upsilon C_{DL} \qquad \text{(S2)}$$

The ECSA value was then determined by correcting C_{DL} with specific capacitance (C_s) which is 0.04 mF cm^{-2} in alkaline solutions.[3]

$$ECSA = \frac{C_{DL}}{C_s} \qquad \text{(S3)}$$

Faradaic efficiency

The produced hydrogen gas during HER is determined by gas chromatography with a thermal conductivity detector (GC-TCD). Pure Ar gas (HP 99.99%) was purged for 30 min before electrolysis. First a calibration curve relating to H_2 concentration was plotted by analysing three GC chromatographs acquired from the real-time quantitative measurement of the gas product during HER in a two-compartment gastight H-cell. The hydrogen gas produced during chronopotentiometry reactions at three current densities of -10, -50, and -100 mA cm^{-2}, each reaction for 1000 s, was carried out by Ar gas flow from reaction cell to GC. Then, the

corresponding faradaic efficiency (FE) at various current densities was calculated according to the following formula:[4]

$$FE = \frac{\alpha F n}{Q} = \frac{\alpha F n}{I_{total}.t} \quad \text{(S4)}$$

$$FE = \frac{(0.1315)V.\upsilon}{I_{total}.t} \times 100 \quad \text{(S5)}$$

where V is Ar gas flow rate (20 mL min^{-1}), ν is the volume concentration of H_2 gas generated in the H-cell, and I is current in A.

Turnover frequency (TOF) calculations

TOF values per Ni active site at surface are calculated based on the following equation according to a previous method.[5]

$$TOF = \frac{Total\ H_2\ turnover\ (cm^2 geometric\ surface\ area)}{Number\ of\ surface\ active\ sites\ (cm^2 geometric\ surface\ area)} \quad \text{(S6)}$$

The amount of total hydrogen gas turnover is obtained with a given current density from polarization curves according to the following equation.

$$H_2\ TN = \left(j\,\frac{mA}{cm^2}\right)\left(\frac{\frac{1C}{s}}{1000\ mA}\right)\left(\frac{1\ mol\ e^-}{96485.3\ C}\right)\left(\frac{1\ mol\ H_2}{2\ mol\ e^-}\right)\left(\frac{6.02\times10^{23} molecules\ H_2}{1\ mol\ H_2}\right) = 3.12 \times 10^{15}\ \frac{H_2/s}{cm^2}\ per\ \frac{mA}{cm^2} \quad \text{(S7)}$$

Assuming that all of Ni sites at the surface of the prepared catalysts are electrochemically active and having a molar volume of 6.59 cm3 mol^{-1} for Ni, surface active sites as the average amount of atoms per 1 square centimeter are estimated according to the following equation.

$$Surface\ Sites = \left(\frac{1\times6.02\times10^{23}\ atoms}{1\ mol}\right) \times \left(\frac{1\ mol}{6.59\ cm^3}\right)^{2/3} = 2.03 \times 10^{15}\ \frac{atoms}{cm^2} \quad \text{(S8)}$$

Afterwards, TOF values can be calculated at a given current density, as following.

$$TOF = \frac{(3.12\times10^{15}\frac{\frac{H_2}{s}}{cm^2}\,per\,\frac{mA}{cm^2})\times|j|}{(2.03\times10^{15}\frac{atoms}{cm^2})\times ECSA} \quad \text{(S9)}$$

Density functional theory (DFT) calculations

Theoretical simulations are carried out using ab initio total energy calculations based on DFT within VASP framework (Vienna Ab initio Simulation Packages) code[6,7], with a plane wave basis set for the electronic orbitals. The electronic exchange and correlation was described within the generalized gradient approximation using the Perdew-Burke-Ernzerhof (PBE) functional[8]. The interaction of the valence electrons with the ionic cores was treated within the projector augmented-wave (PAW) method[9]. A Monkhorst-Pack grid was used by K-points 3x3x1[10]. The employed cut-off energy was set as 450 eV. An electronic smearing was introduced within the Methfessel-Paxton scheme with N = 0 and σ = 0.1 eV[11]. Spin-polarization effect was considered. A large supercell of 11.37894 Å x 11.37894 Å x 24.00 Å was modelled for describing the geometric structures. To take the dispersion forces into account, van der Waals correlation function was considered in this work. All DFT calculations were performed to minimize all the residual forces until the convergence criterion of 0.01 eV/Å. The central difference was used to calculate the zero-point energy (ZPE) such that the positions of mobile atoms (*i.e.*, *H species) are displaced in each direction by a small positive and negative displacement as 0.01 Å. Considering the contributions from the energy change, the difference of zero-point energy and entropy contribution, respectively, the adsorption free energy is given by

$$\Delta G(H^*) = \Delta E + \Delta ZPE - T\Delta S \quad \text{(S10)}$$

Zero-point energy correction (ΔZPE) for various absorbents and TΔS at 298 K are adapted. Thus, referring to H_2 (gas) free energy[12], the binding energy is given by

$$\Delta E = E\left(\frac{H^*}{slab}\right) - E(slab) - 0.5 \times E(H_2) \quad \text{(S11)}$$

where E(*H/slab) and E(slab) are the total energies of *H species adsorption on the slab and the clean slab. Similarly, the adsorption of *OH on the Ni top sites and the co-adsorption of *H-*OH on the Ni bridge and top sites, respectively, in NiN_3-C phase in $Ni(OH)_2$@NiN_3-C nano-heterostructure are calculated according to following definitions of Gibbs adsorption free energy:

$$\Delta G(*OH) = \Delta G(*OH/Slab) - \Delta G(Slab) - \Delta G(H_2O) + 0.5*\Delta G(H_2) \quad \text{(S12)}$$

$$\Delta G(*H\text{-}*OH) = \Delta G(*H\text{-}*OH/Slab) - \Delta G(Slab) - \Delta G(H_2O) \quad \text{(S13)}$$

where ΔG(*OH/Slab) and ΔG(*H-*OH/Slab) are the Gibbs adsorption free energy of *OH and *H-*OH adsorbates. The values of ΔG(Slab), $\Delta G(H_2O)$ and $\Delta G(H_2)$ are the Gibbs adsorption free energy of the substrate, water and hydrogen molecules, respectively. The zero-point energy and entropy contributions are also considered for H_2 and H_2O molecules as well as for the adsorption of *OH and the co-adsorption of *H-*OH species.

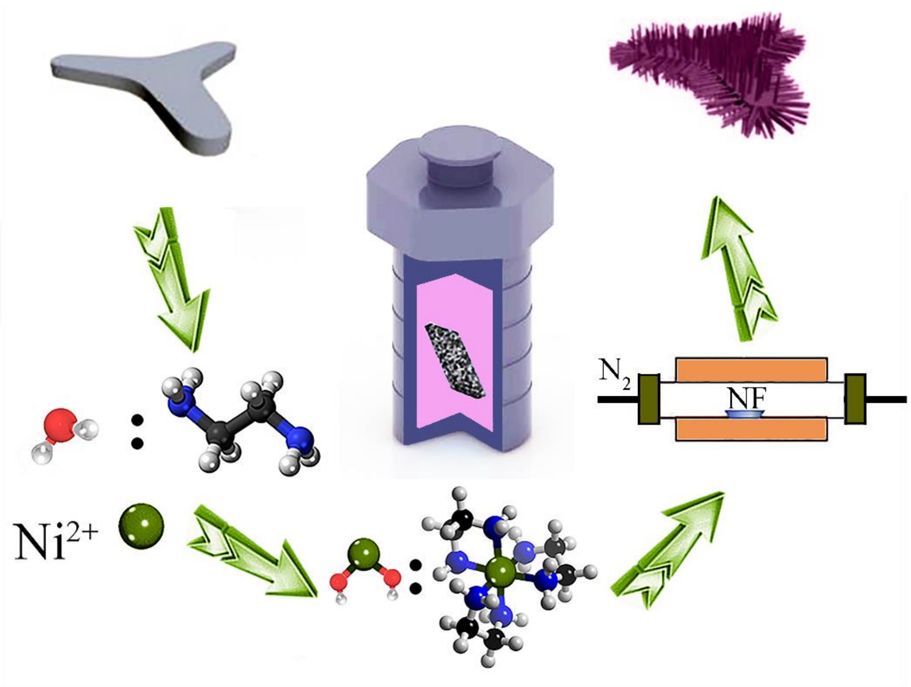

Figure S1. Schematic representing the synthesis procedure using solvothermal reaction of metallic Ni foam as starting material and post-thermal treatment in nitrogen atmosphere.

Supplementary Note 1

Solvothermal optimization by RSM modelling

The required overpotential to deliver a HER current density of -100 mA cm^{-2} is used as output in the RSM model. Four control parameters including the concentration of added Ni ions, volume ratio of H_2O:SDA solution (SDA: structure directing agent), solvothermal time and temperature are used as input. A two-term hypothesis based on first degree and quadratic terms is built to take the interactions of the inputs (**Figure S2**). The simulated surface response models demonstrate the variation of two control parameters whilst the other two are kept at their predicted optimums (**Figure S3**). Considering the first-degree terms, solvothermal temperature imposes the biggest effect, resulting in tilted surface. Whereas the quadratic terms, representing the mutual interactions between control factors, indicate more weight for solvothermal time which gives a concave function to the surface response. Optimal conditions are selected as 5 mM Ni^{2+}, a H_2O:SDA ratio of 3:2 (SDA: structure directing agents) as well as solvothermal time and temperature of 5 h and 180°C. With increasing the concentration of Ni ions and H_2O:SDA (W/S) volume ratio, self-crystallization to $Ni(en)_3]^{2+}$ complex happens, providing Ni-N/Ni-C moieties on surface. However, molecular $Ni(en)_3]^{2+}$ deteriorates with increasing the HER potential. Therefore, 5 mM and 3:2 volumetric ratio were opted as predicted optimums.

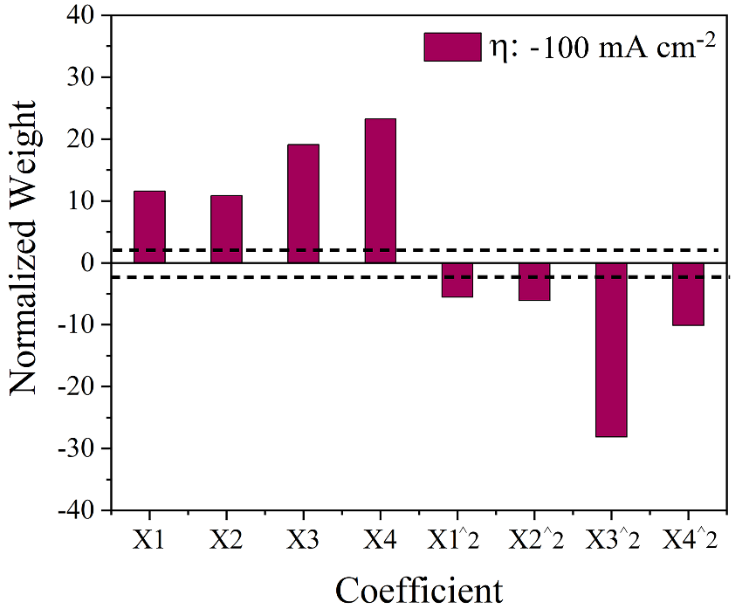

Figure S2. Contribution of the first degree and quadratic terms and their interaction on control parameters in the solvothermal reaction.

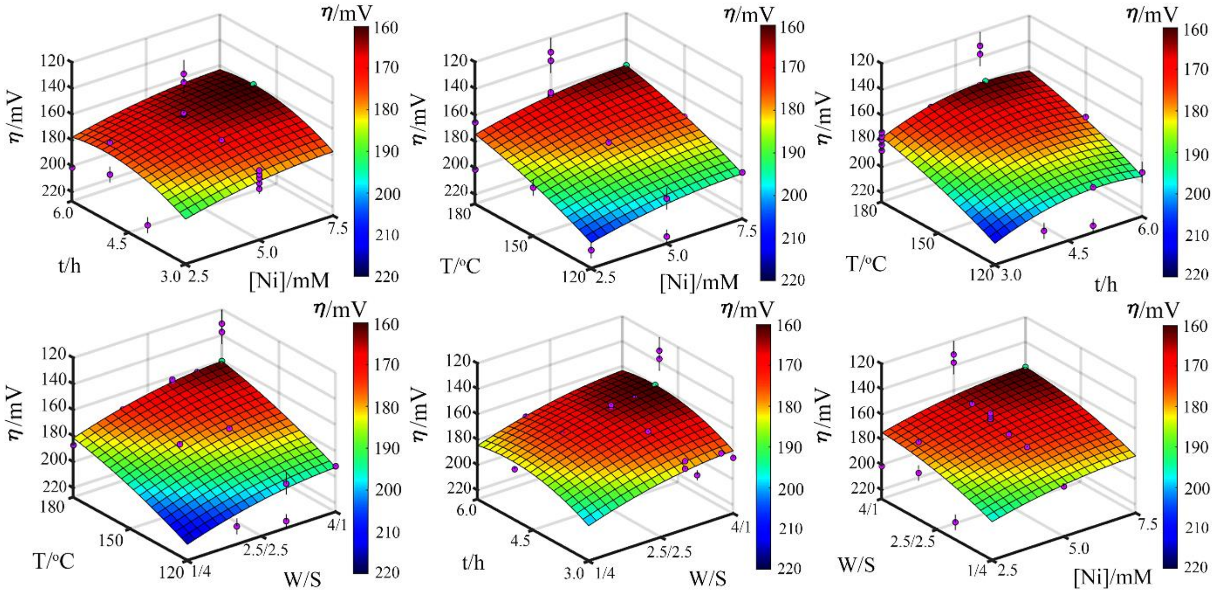

Figure S3. RSM surface models of control parameters for fabricating $Ni(OH)_2$@Ni-N/Ni-C/NF. Experimental (pink) and predicted optimum (green) are captured at -100 mA cm^{-2}.

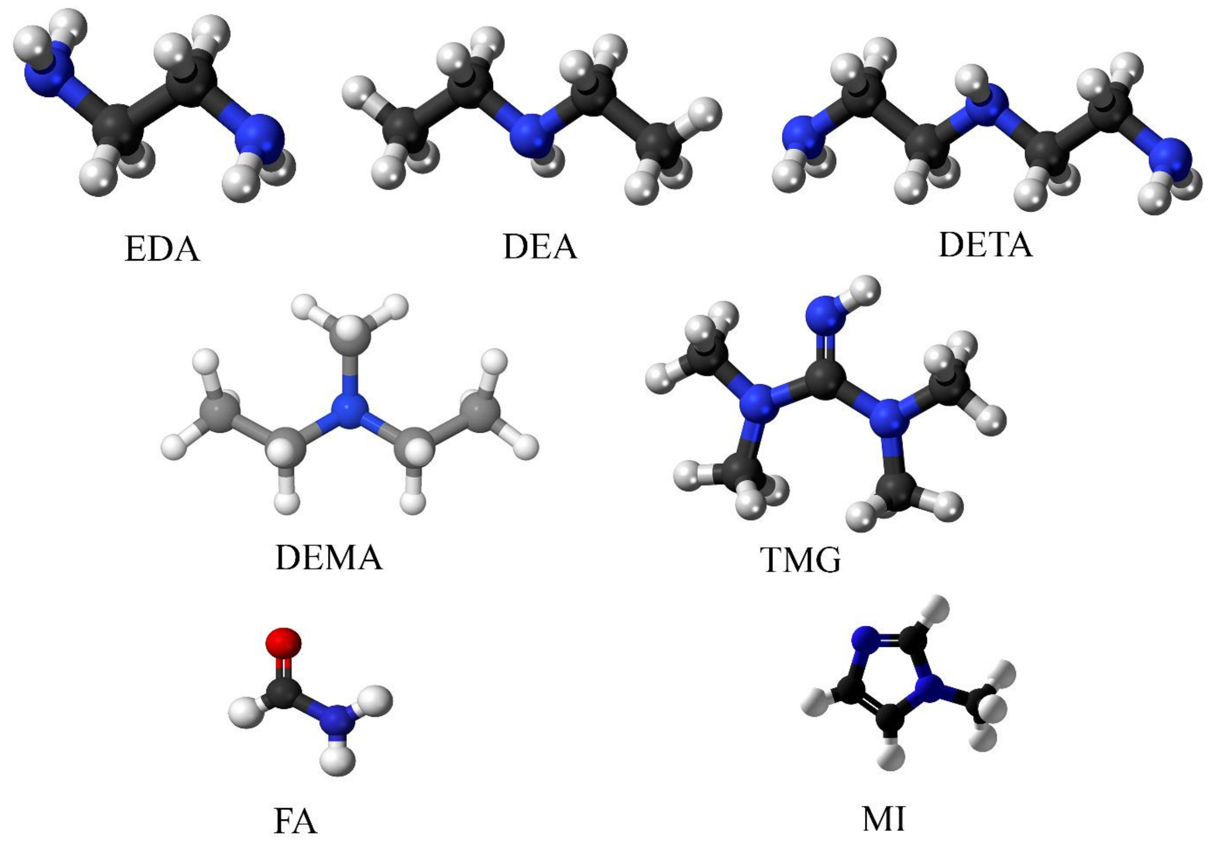

Figure S4. N-containing SDAs to construct hetero-hierarchical nanostructures at NF surface.

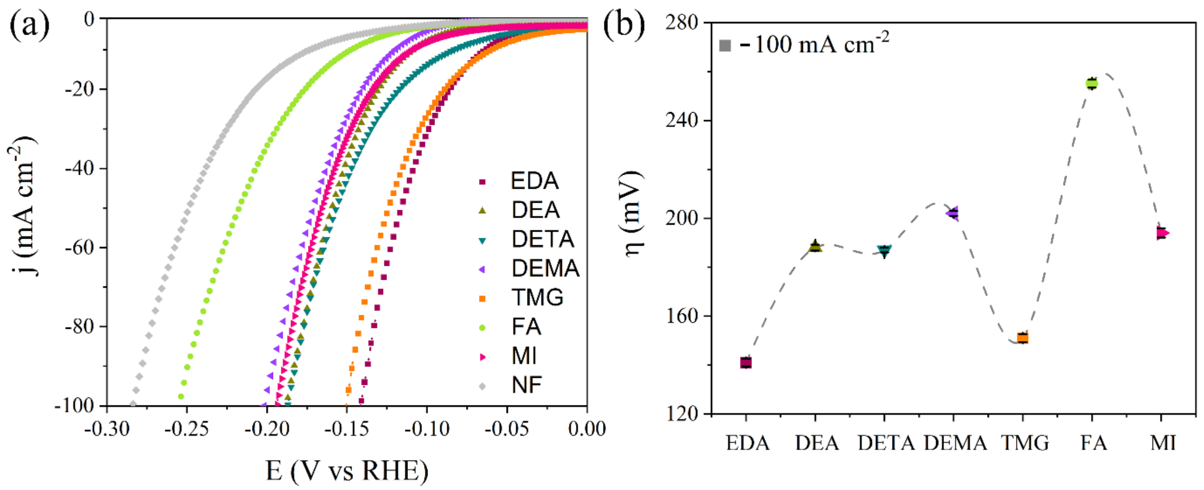

Figure S5. Effect of SDAs on HER. (a) LSV curves at 90% ohmic compensation level. (b) The corresponding overpotential values at a current density of -100 mA cm^{-2}.

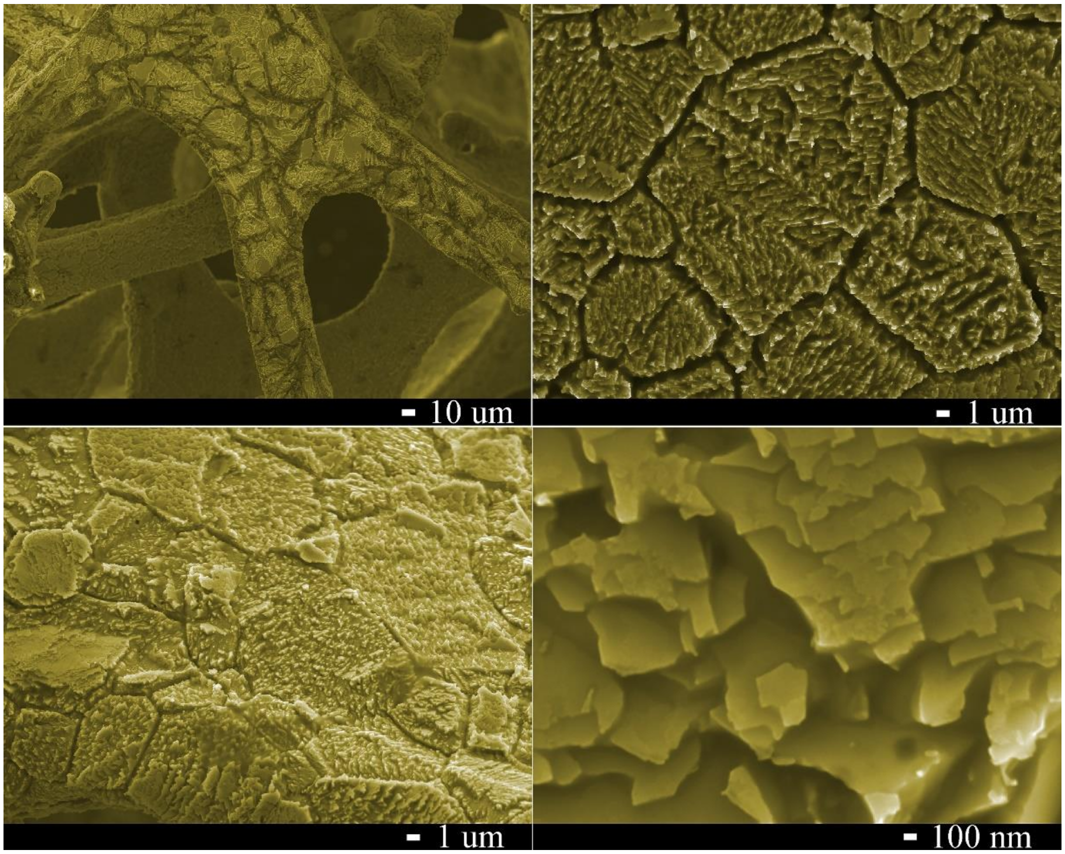

Figure S6. SEM micrographs of $Ni(OH)_2$@Ni-N/Ni-C/NF with different directional hierarchical zones.

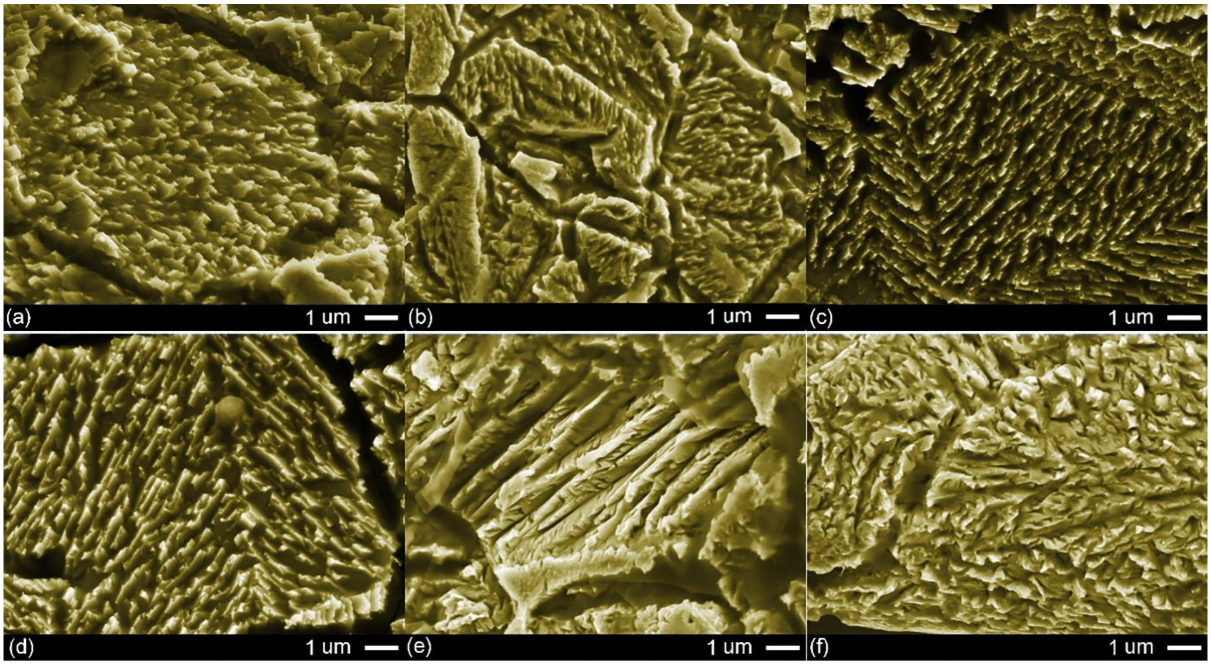

Figure S7. Hetero-hierarchical nanostructure evolution guided by RSM modelling. (a) t: 3 h and [Ni]: 5 mM. (b) T: 150° and [Ni]: 5 mM. (c) Optimal parameters, T: 180° and t: 5 h. (d) T: 150° and W/S: 4/1. (e) t: 3 h and W/S: 1/4. (f) W/S: 1/4 and [Ni]: 5 mM.

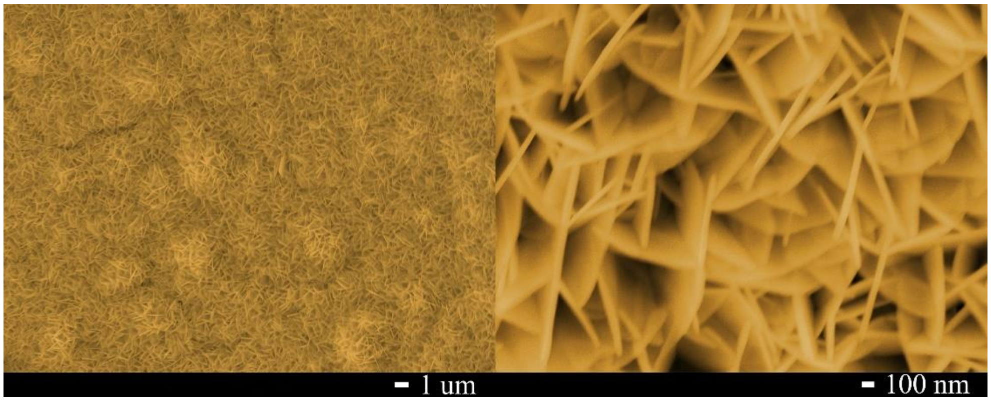

Figure S8. SEM image of micro and nanosheets on $Ni(OH)_2$/NF.

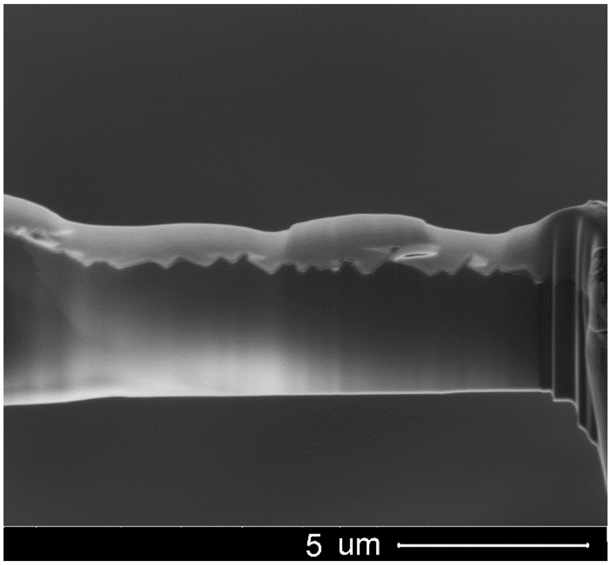

Figure S9. FIB-SEM image of surface hierarchies on $Ni(OH)_2$@Ni-N/Ni-C/NF with deposited Pt atop.

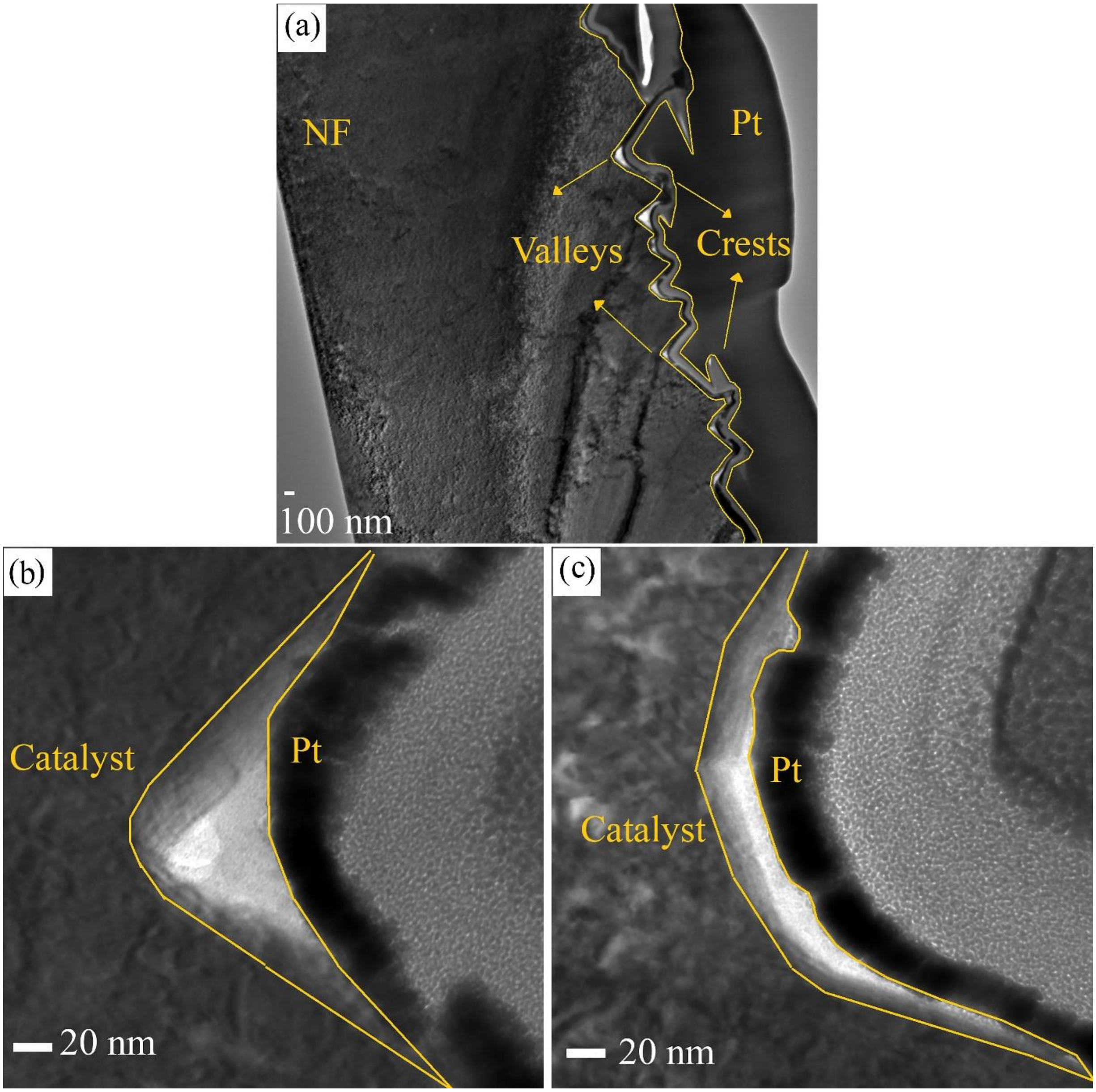

Figure S10. (a) High as well as (b) and (c) low resolution FIB-STEM image of $Ni(OH)_2$@Ni-N/Ni-C/NF with deposited Pt atop.

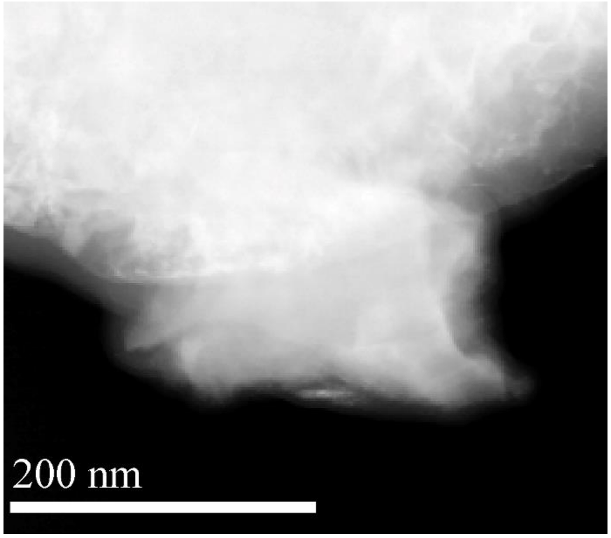

Figure S11. HAADF-STEM image of $Ni(OH)_2$@Ni-N/Ni-C/NF.

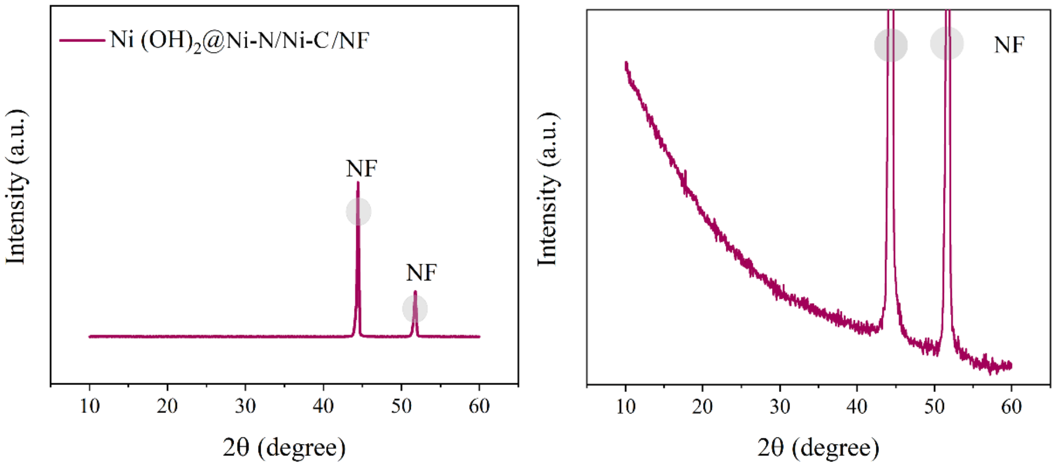

Figure S12. GIXRD pattern of $Ni(OH)_2$@Ni-N/Ni-C/NF.

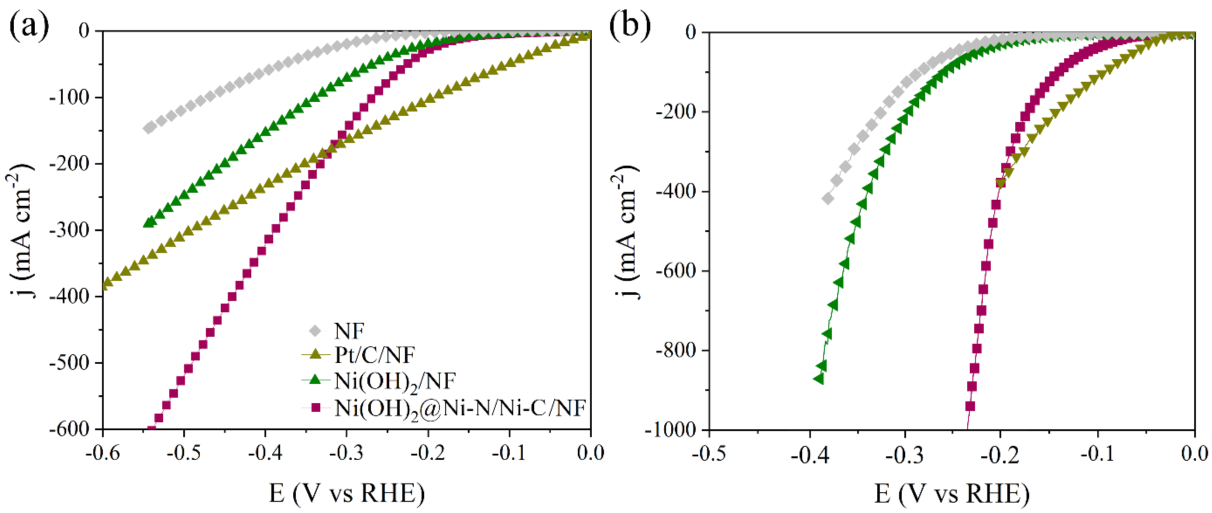

Figure S13. Polarization curves (a) without and (b) with 90% ohmic resistance compensation.

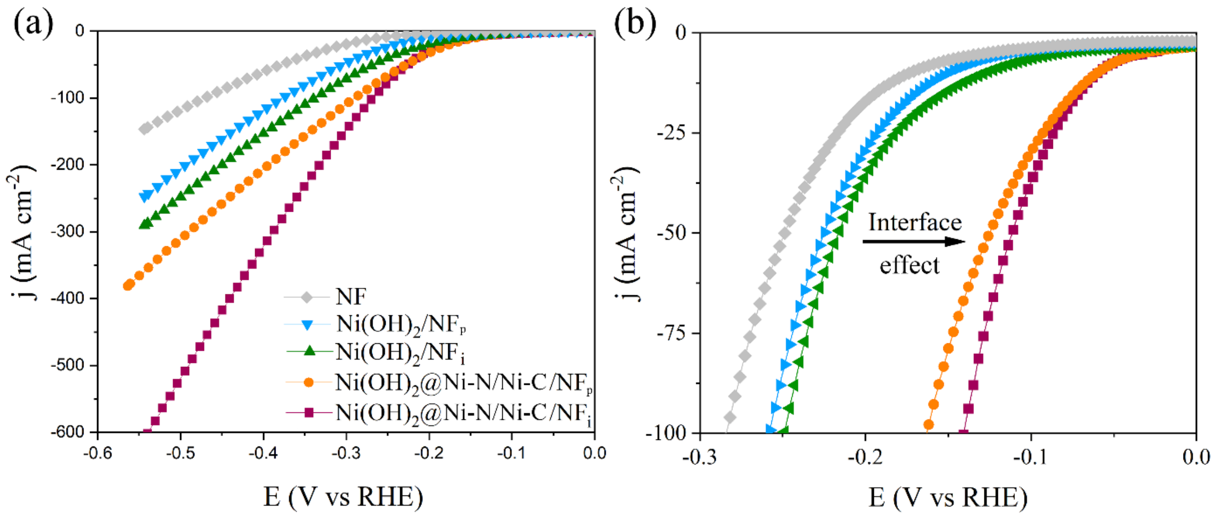

Figure S14. LSV curves of monophasic and nano-heterostructure Ni electrodes (a) without and (b) with 90% ohmic resistance compensation.

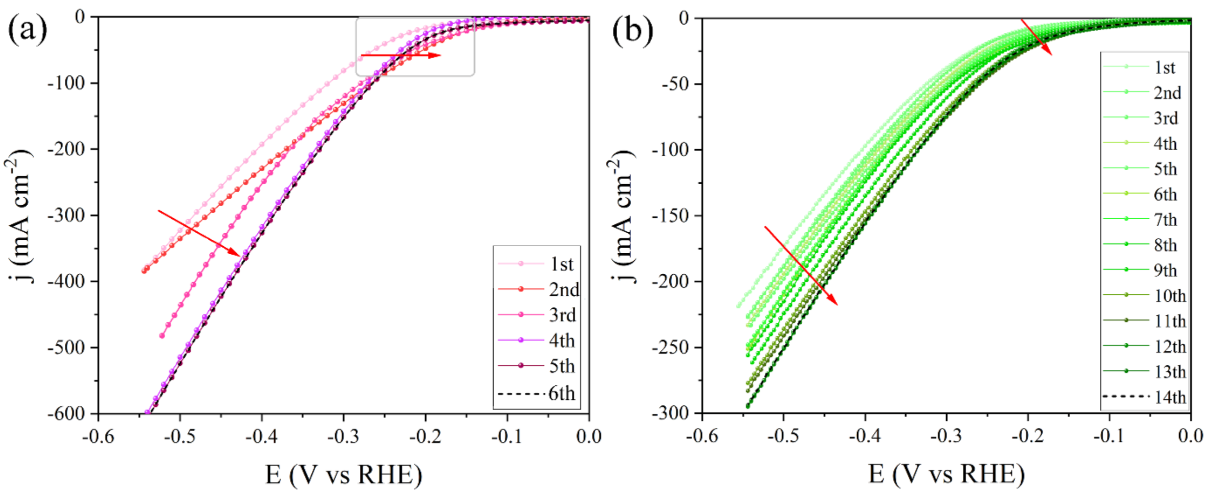

Figure S15. Stabilization of LSV curves for (a) $Ni(OH)_2$@Ni-N/Ni-C/NF and (b) $Ni(OH)_2$/NF at 5 mV s^{-1} in 1 M KOH.

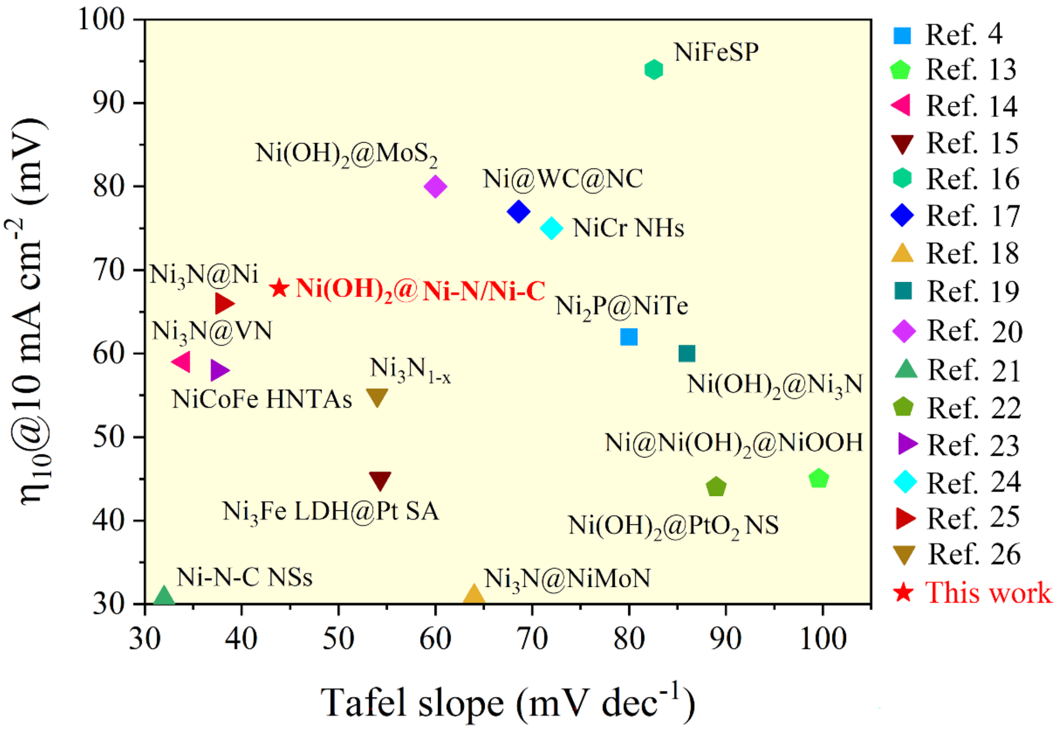

Figure S16. Electrocatalytic performance of the developed $Ni(OH)_2$@Ni-N/Ni-C compared to the best Ni-based multimetallic and multiphasic catalysts for alkaline HER.[4,13-26]

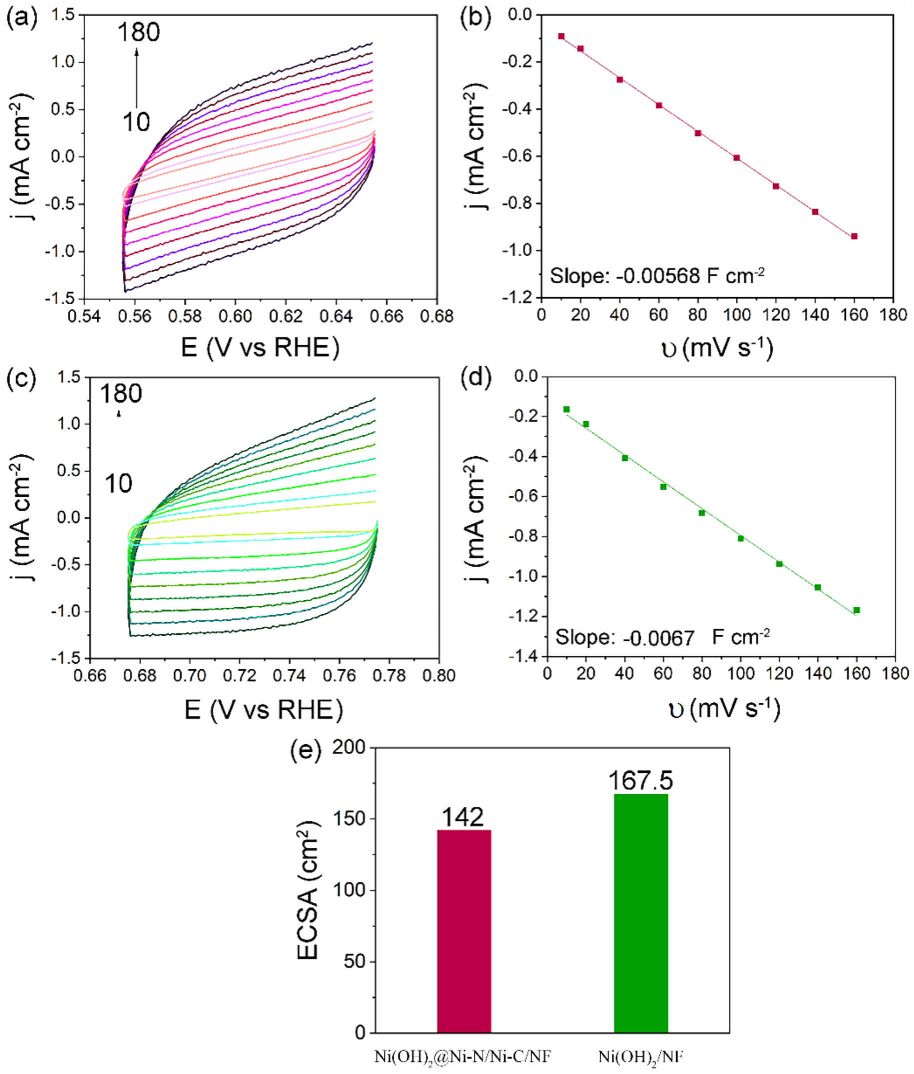

Figure S17. CV curves and current-scan rate linear regressions for (a) and (b) $Ni(OH)_2$@Ni-N/Ni-C/NF, as well as (c) and (d) $Ni(OH)_2$/NF, along with (e) the corresponding ECSA values.

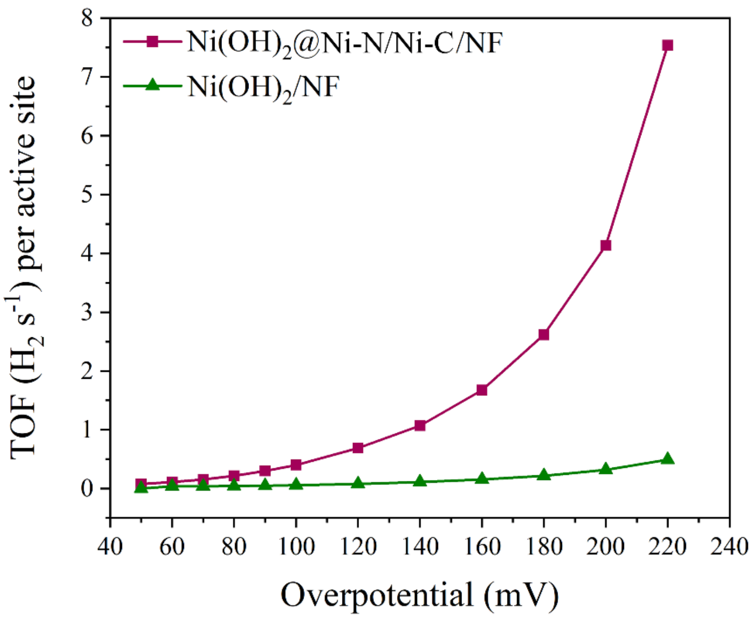

Figure S18. Trends of TOF values of $Ni(OH)_2$@Ni-N/Ni-C/NF and $Ni(OH)_2$/NF with increasing HER overpotential.

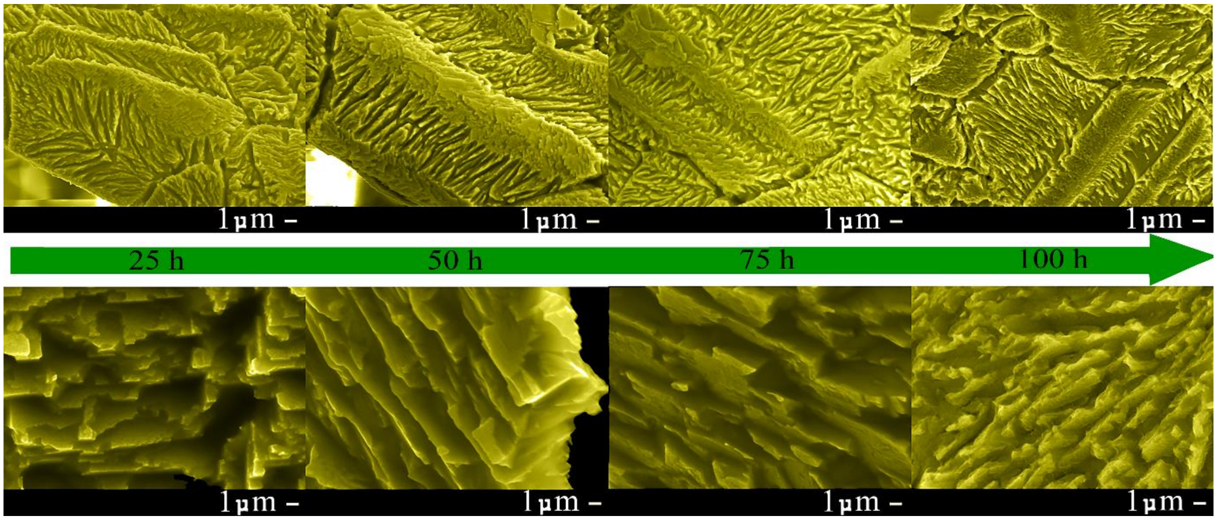

Figure S19. SEM micrographs of $Ni(OH)_2$@Ni-N/Ni-C/NF during HER at -100 mA cm^{-2}.

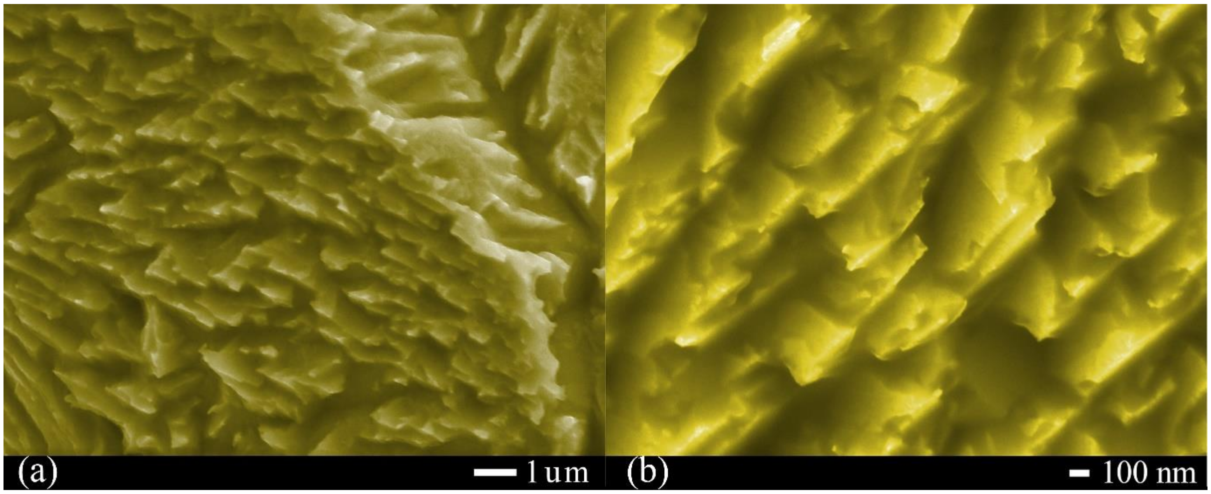

Figure S20. SEM micrographs of $Ni(OH)_2$@Ni-N/Ni-C/NF during HER at -500 mA cm^{-2}.

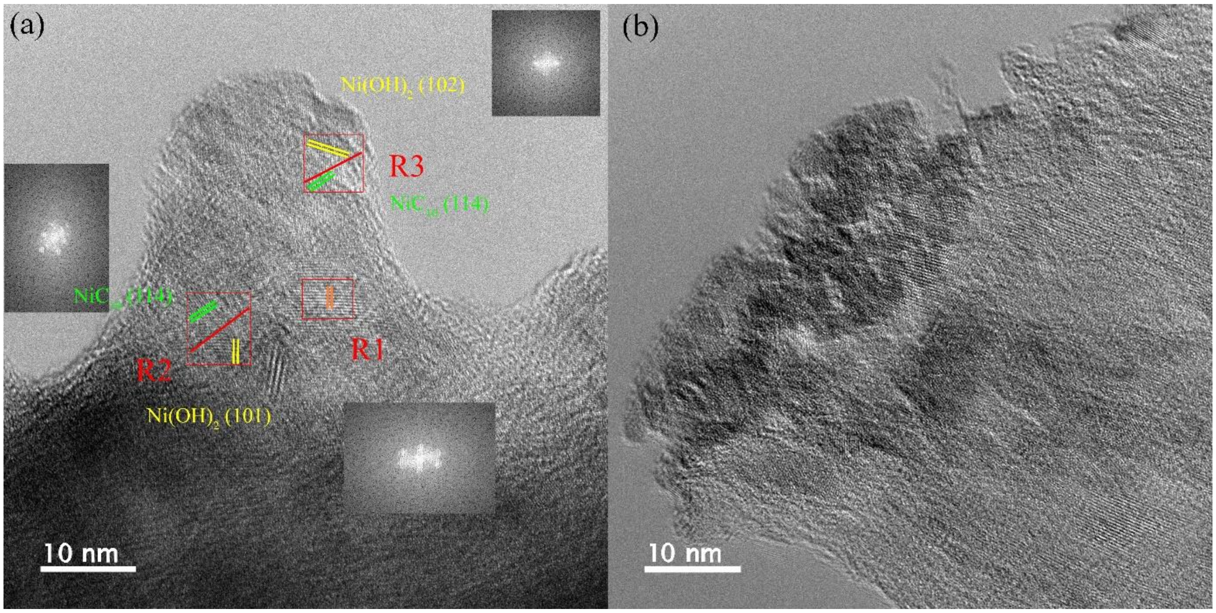

Figure S21. HRTEM images of Ni(OH)$_2$@Ni-N/Ni-C/NF at (a) 10 h and (b) 100 h of HER.

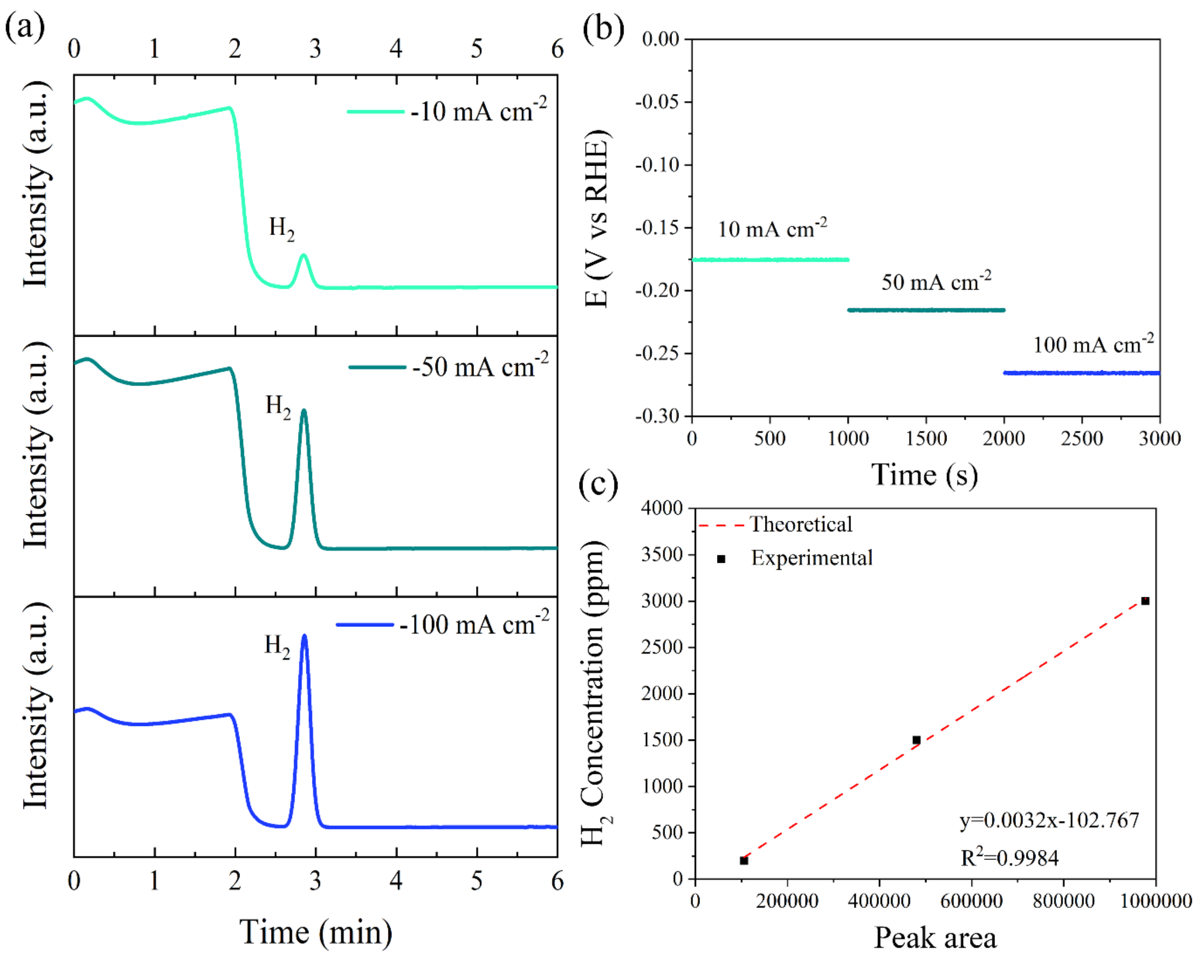

Figure S22. HER Faradaic efficiency using Ni(OH)$_2$@Ni-N/Ni-C/NF. (a) GC-TCD chromatograms of (b) step-chronopotentiometry at different HER current densities. (c) Theoretical vs. experimental amounts of H_2 generated at these current densities.

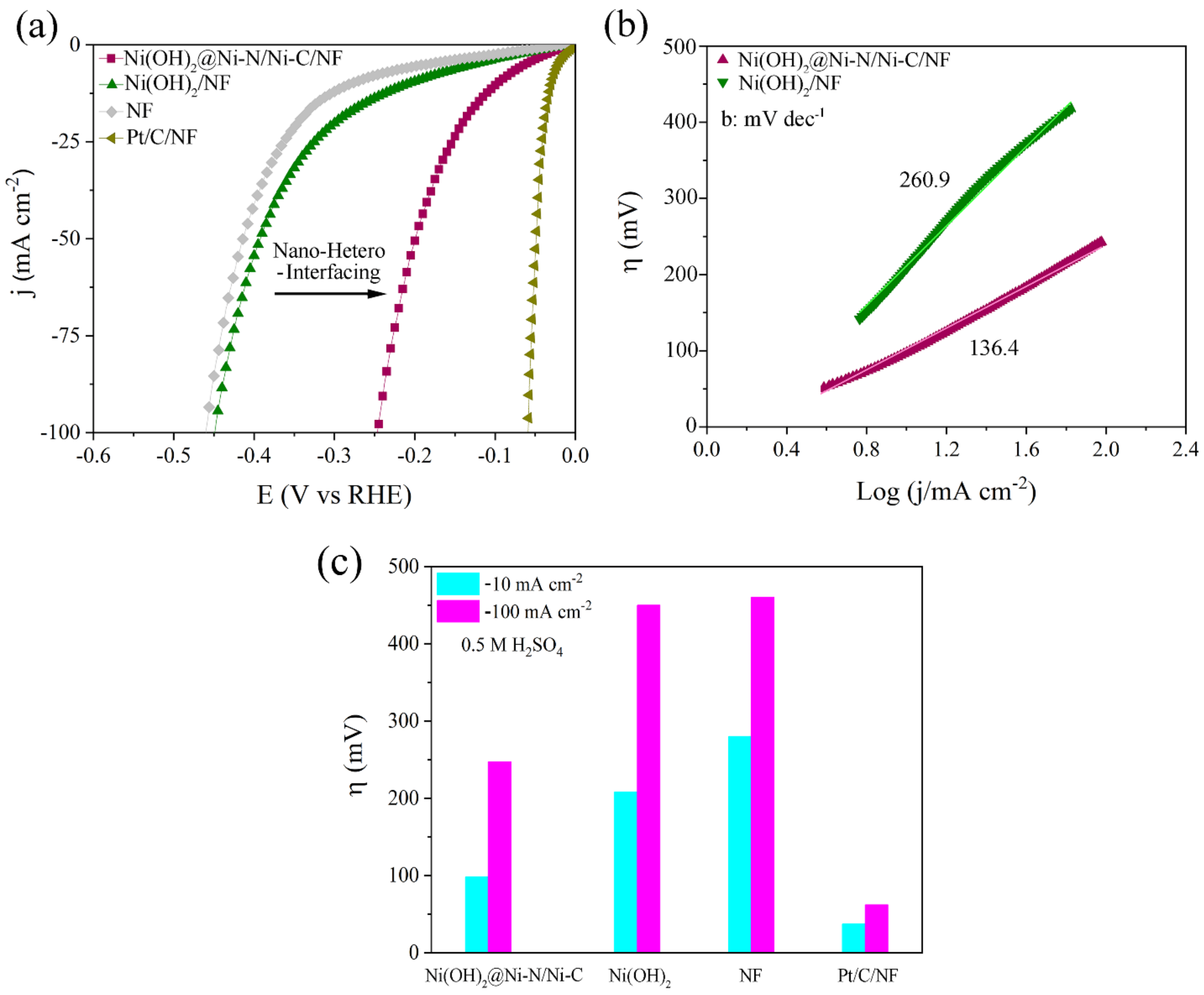

Figure S23. (a) HER polarization curves of bare Ni foam, $Ni(OH)_2$/NF, $Ni(OH)_2$@Ni-N/Ni-C/NF, and Pt/C/NF in 0.5 M H_2SO_4. The corresponding (b) Tafel slopes and (c) overpotentials trends.

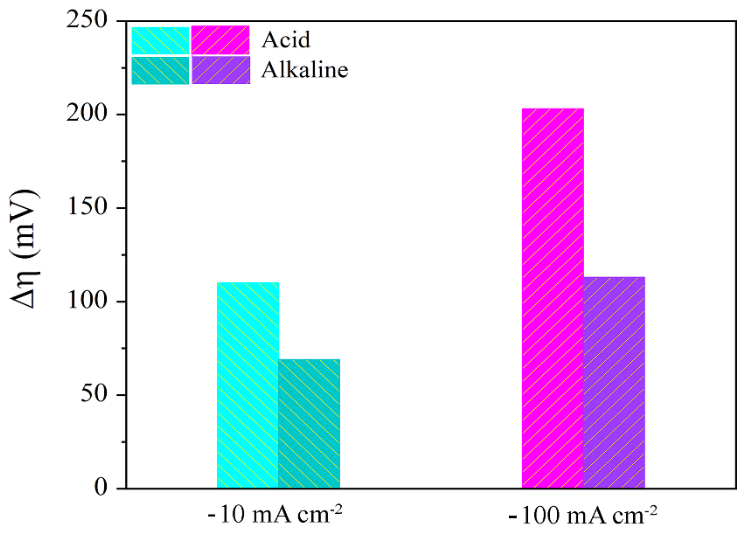

Figure S24. Comparison of the differences in overpotentials derived by $Ni(OH)_2$/NF and $Ni(OH)_2$@Ni-N/Ni-C/NF at -10 and -100 mA cm^{-2} between acidic and alkaline electrolytes.

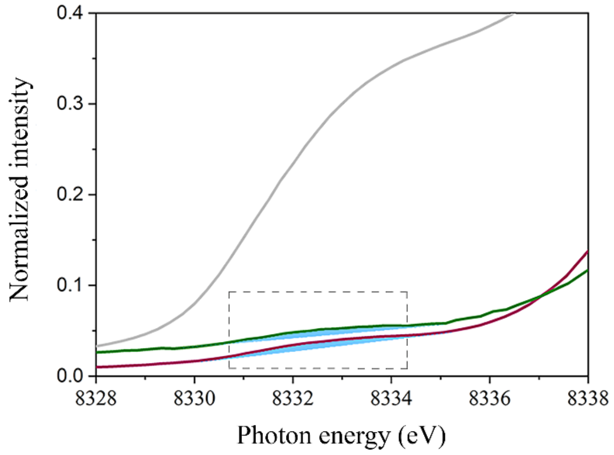

Figure S25. XANES pre-edge of $Ni(OH)_2$@Ni-N/Ni-C and $Ni(OH)_2$.

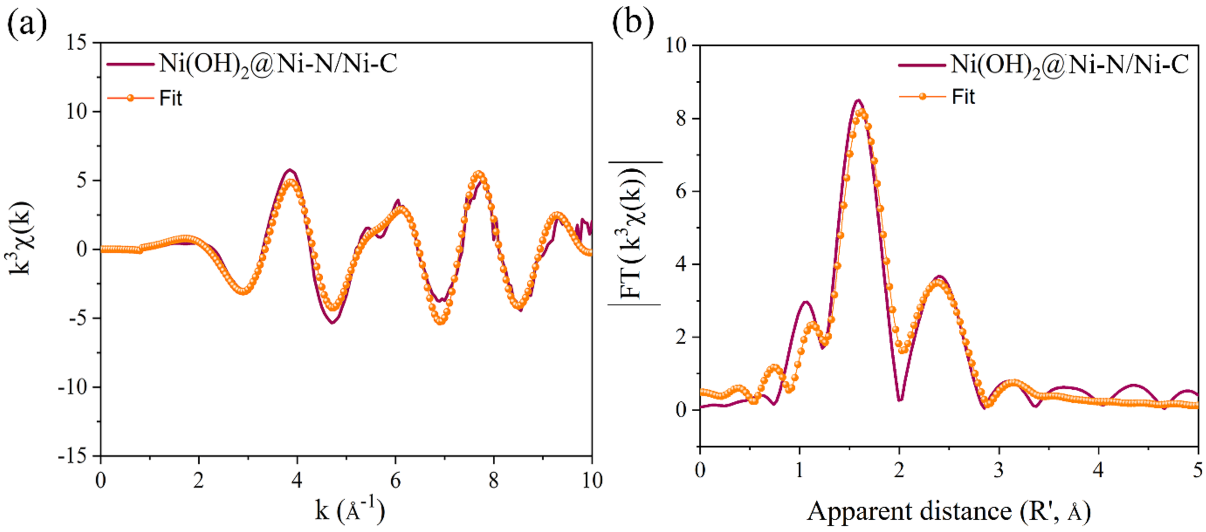

Figure S26. EXAFS fitting of $Ni(OH)_2$@Ni-N/Ni-C/Ni/CFP at (a) k-range and (b) radial space.

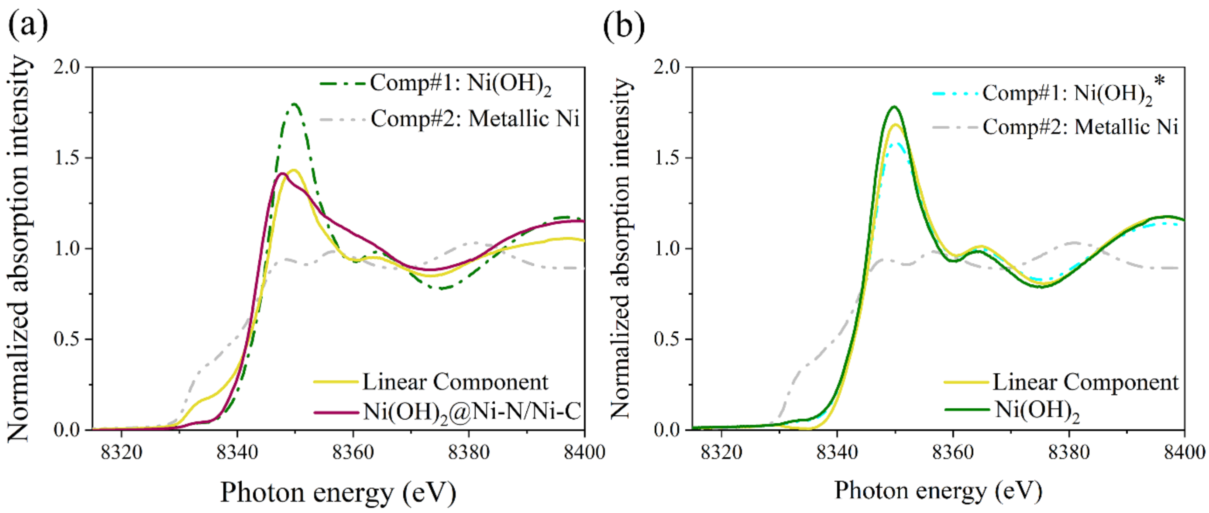

Figure S27. Comparison of the linear combinations with the XANES spectra of (a) $Ni(OH)_2$@Ni-N/Ni-C/Ni/CFP and (b) $Ni(OH)_2$/Ni/CFP.

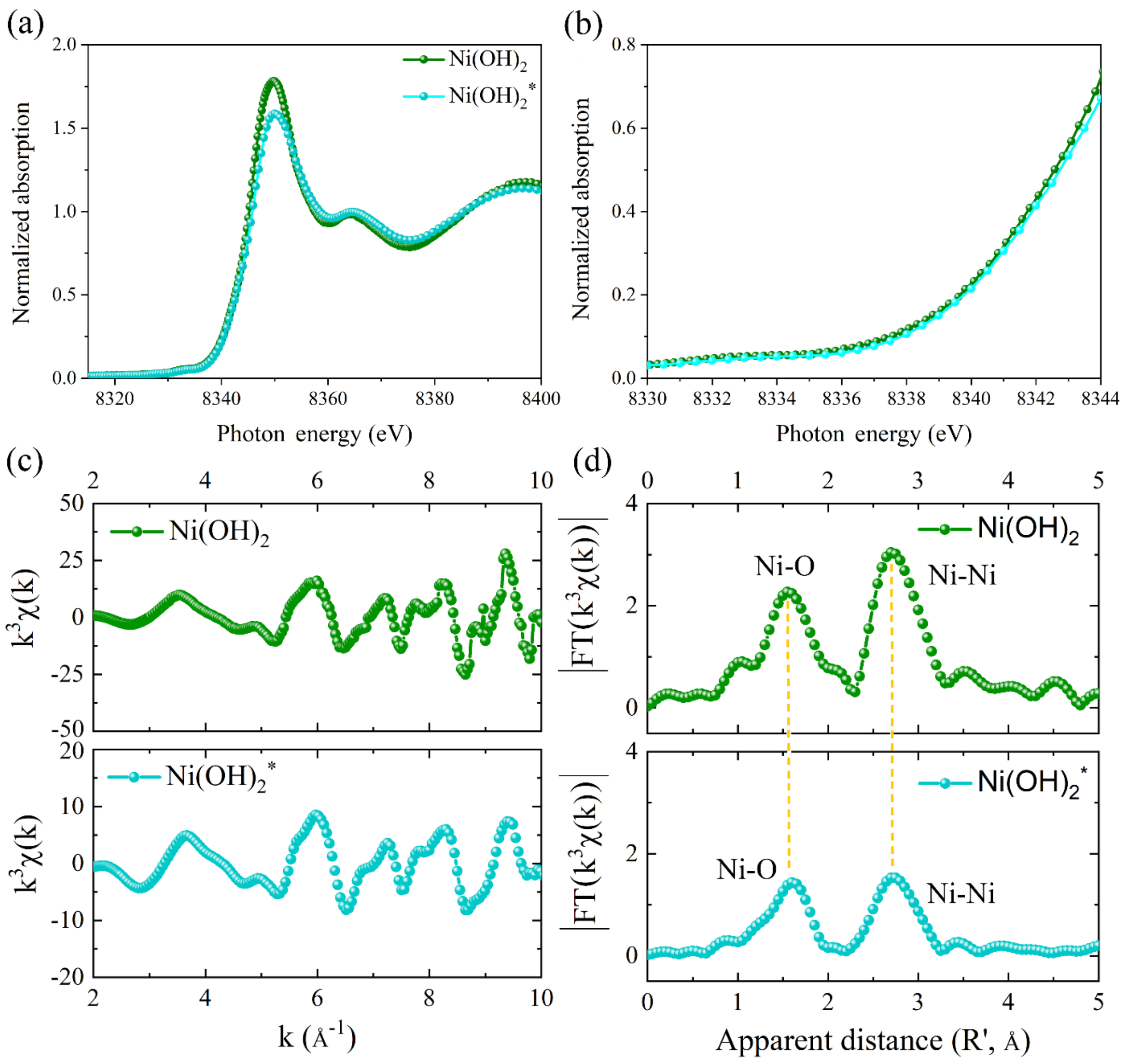

Figure S28. (a) and (b) XANES spectra, (c) Ni k edge EXAFS, and (d) Fourier transforms of EXAFS of synthesized $Ni(OH)_2$ and standard $Ni(OH)_2$*.

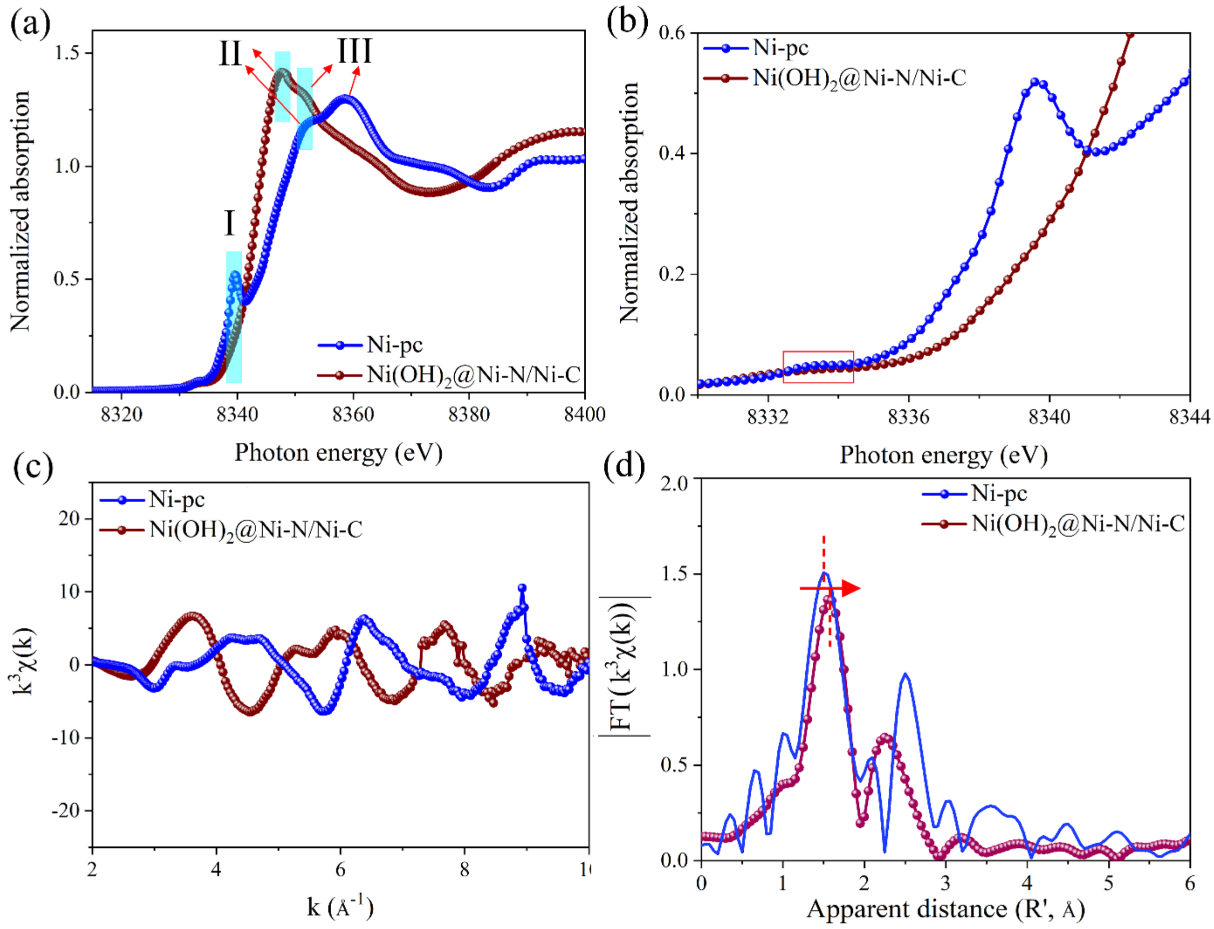

Figure S29. Comparison of (a) and (b) XANES spectra, (c) Ni k edge EXAFS, and (d) Fourier transforms of EXAFS of $Ni(OH)_2$@Ni-N/Ni-C/Ni/CFP against Ni-pc/CFP.

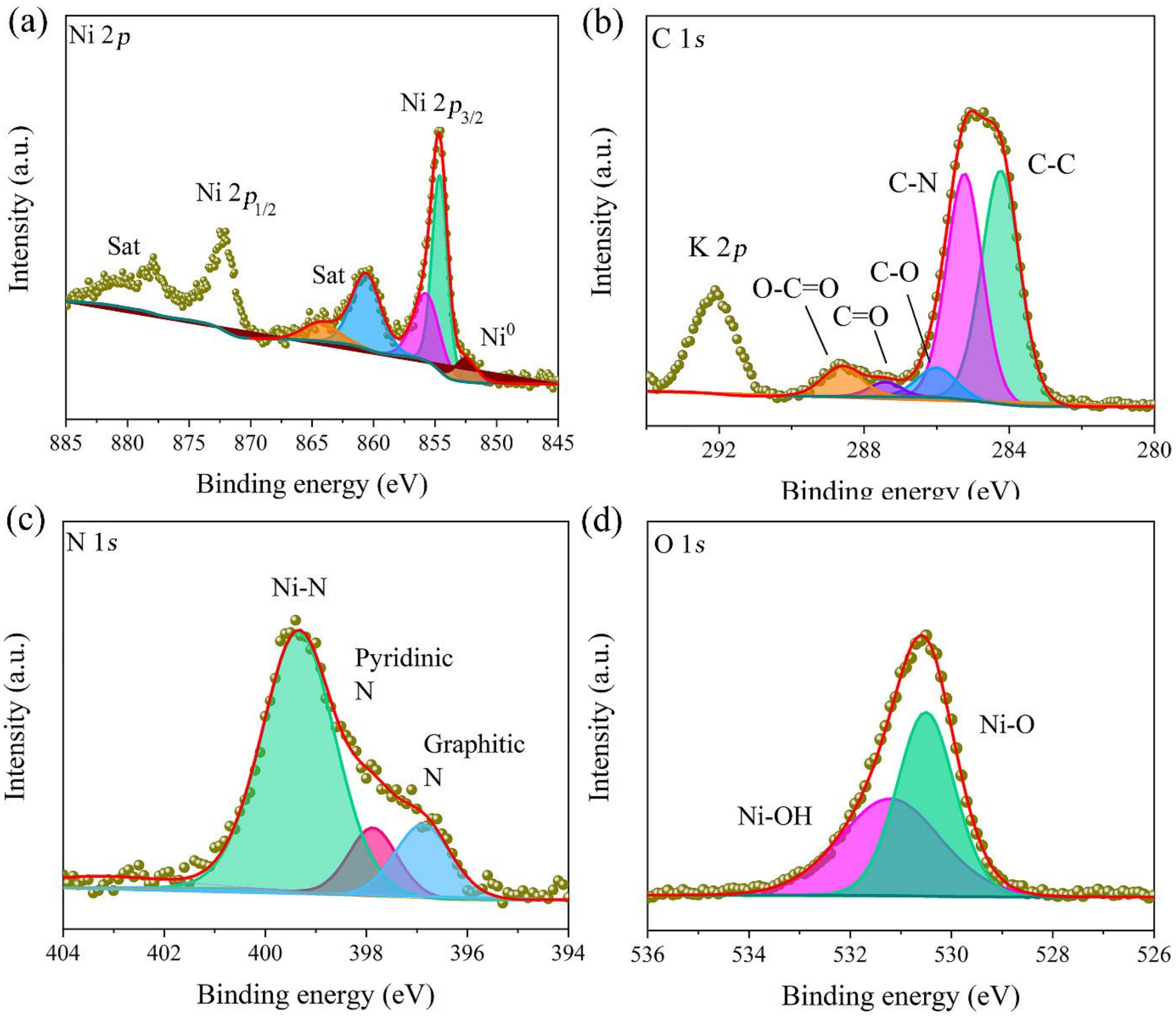

Figure S30. Core-level XPS profiles of (a) Ni 2*p*, (b) C 1*s*, (c) N 1*s*, (d) O 1*s* of $Ni(OH)_2$@Ni-N/Ni-C/NF.

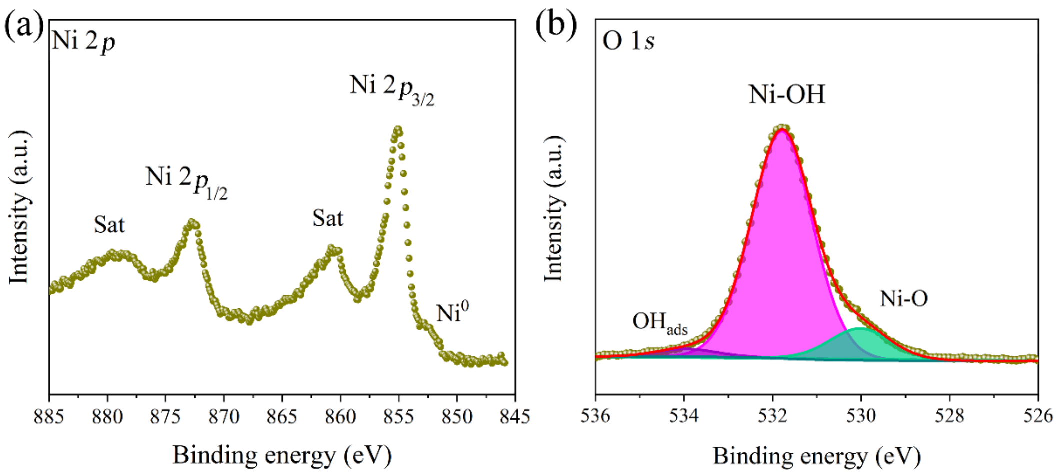

Figure S31. Core-level (a) Ni 2*p* and (b) O 1*s* XPS spectra of $Ni(OH)_2$/NF.

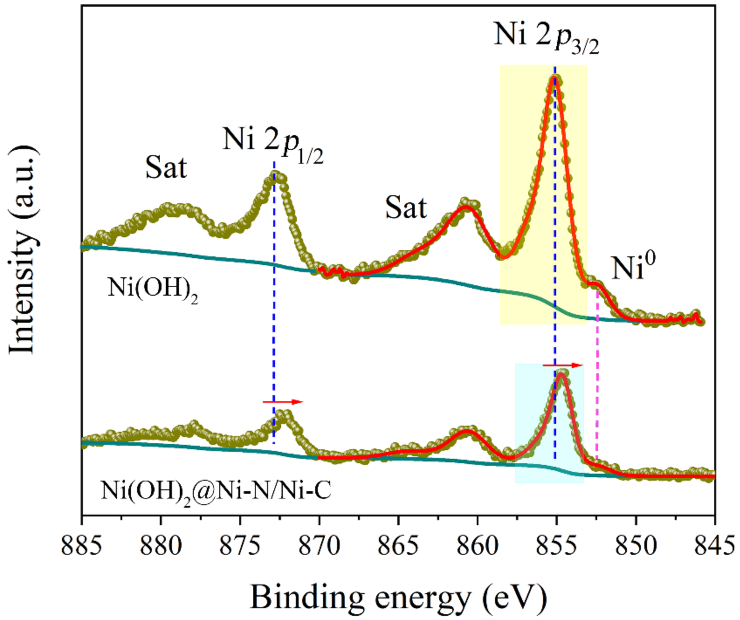

Figure S32. The shift of Ni 2*p* XPS peaks to lower binding energies in $Ni(OH)_2$@Ni-N/Ni-C compared to Ni 2*p* XPS peaks in the monophasic $Ni(OH)_2$.

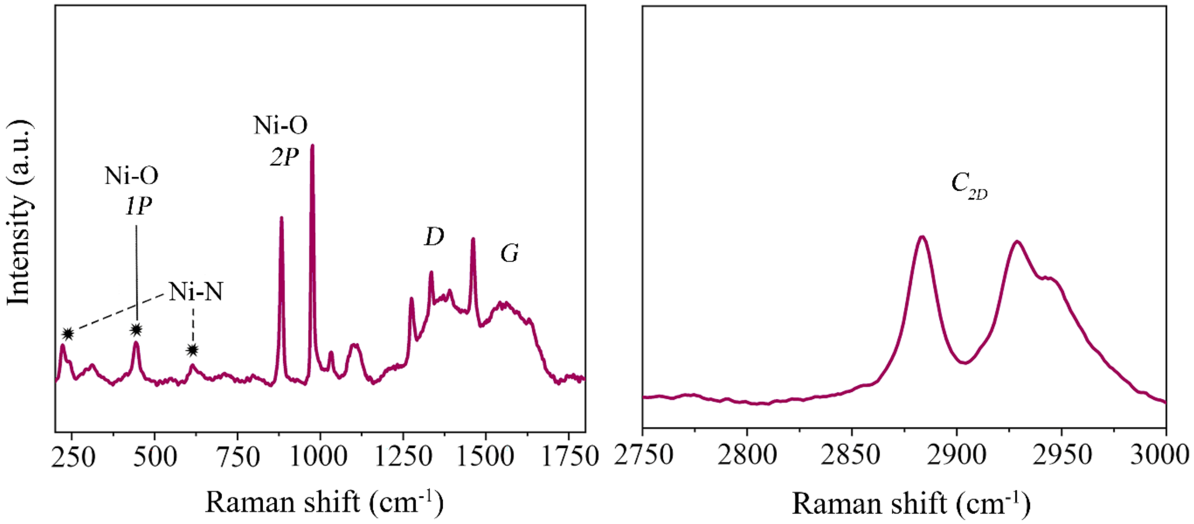

Figure S33. Raman spectrum of $Ni(OH)_2$@Ni-N/Ni-C/NF.

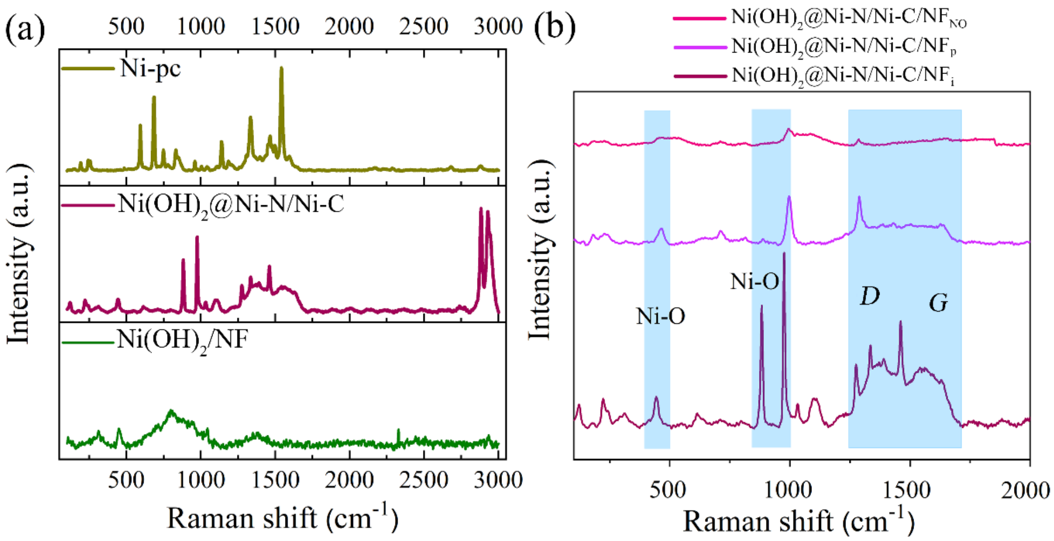

Figure S34. Comparison of Raman spectra and shifts in (a) $Ni(OH)_2$@Ni-N/Ni-C/NF, $Ni(OH)_2$/NF, and Ni-pc, as well as in nano-heterostructure (b) with added Ni ions ($Ni(OH)_2$@Ni-N/Ni-C/NF_i), from pristine Ni foam ($Ni(OH)_2$@Ni-N/Ni-C/NF_p), and non-optimal sample by RSM model ($Ni(OH)_2$@Ni-N/Ni-C/NF_{NO}).

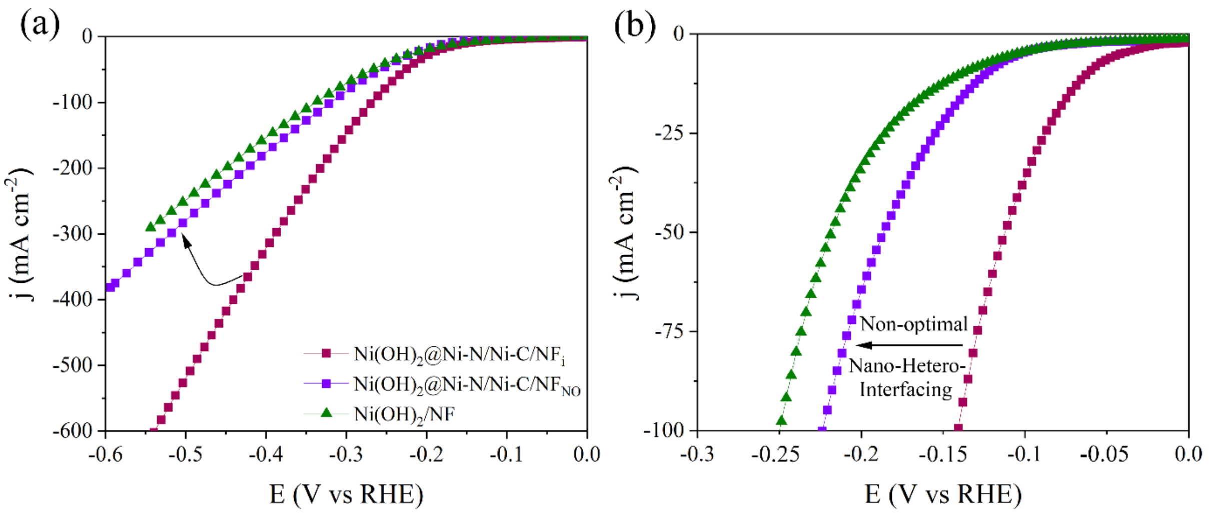

Figure S35. Comparison of HER LSV curves of the optimal $Ni(OH)_2$@Ni-N/Ni-C/NF_i nano-heterostructure with ionic Ni precursor, non-optimal $Ni(OH)_2$@Ni-N/Ni-C/NF_{NO} heterostructure, and monophasic $Ni(OH)_2$/NF in 1 M KOH.

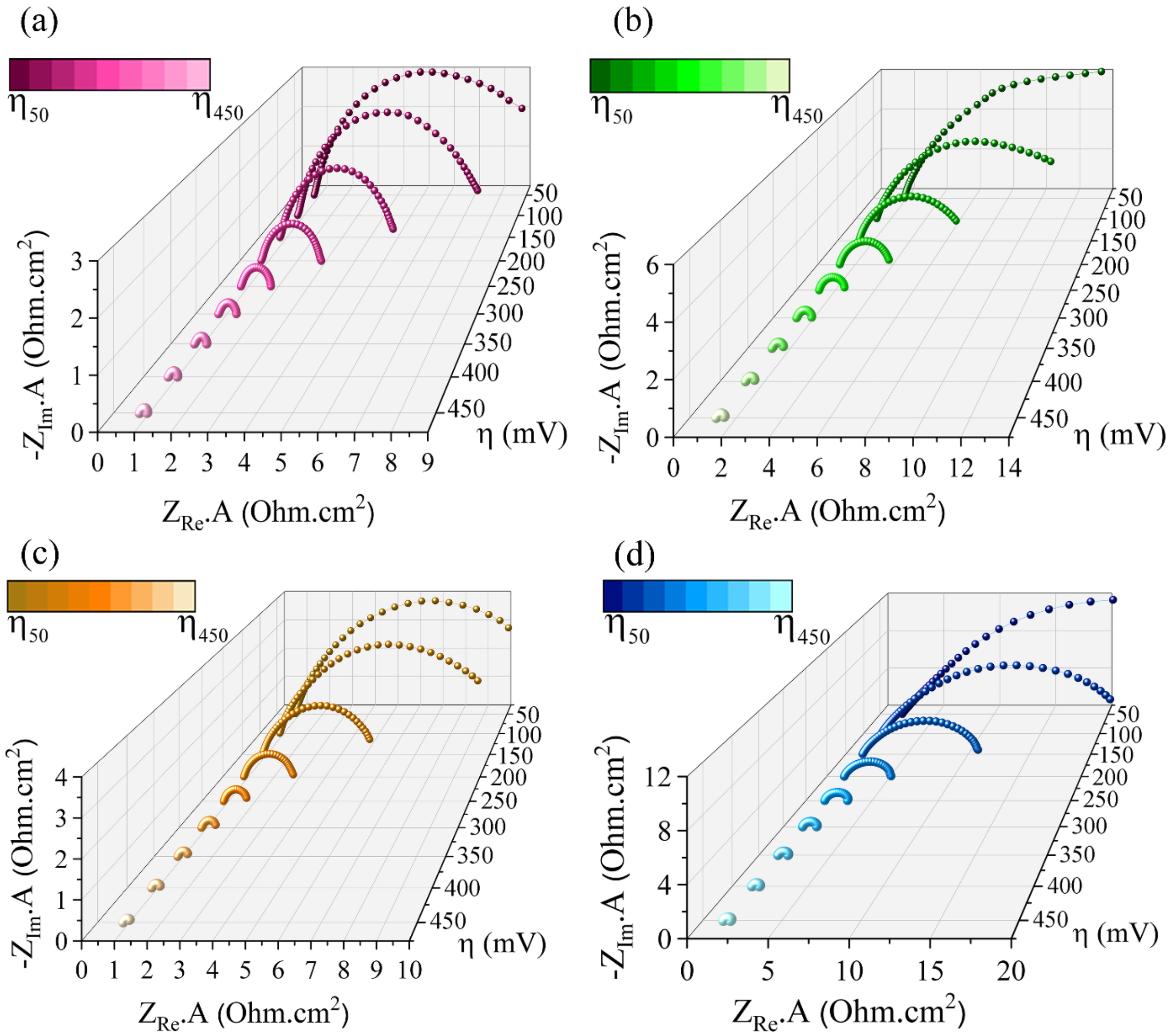

Figure S36. 3D Nyquist plots of (a) $Ni(OH)_2$@Ni-N/Ni-C/NF_i, (b) $Ni(OH)_2/NF_i$, (c) $Ni(OH)_2$@Ni-N/Ni-C/NF_p and (b) $Ni(OH)_2/NF_p$.

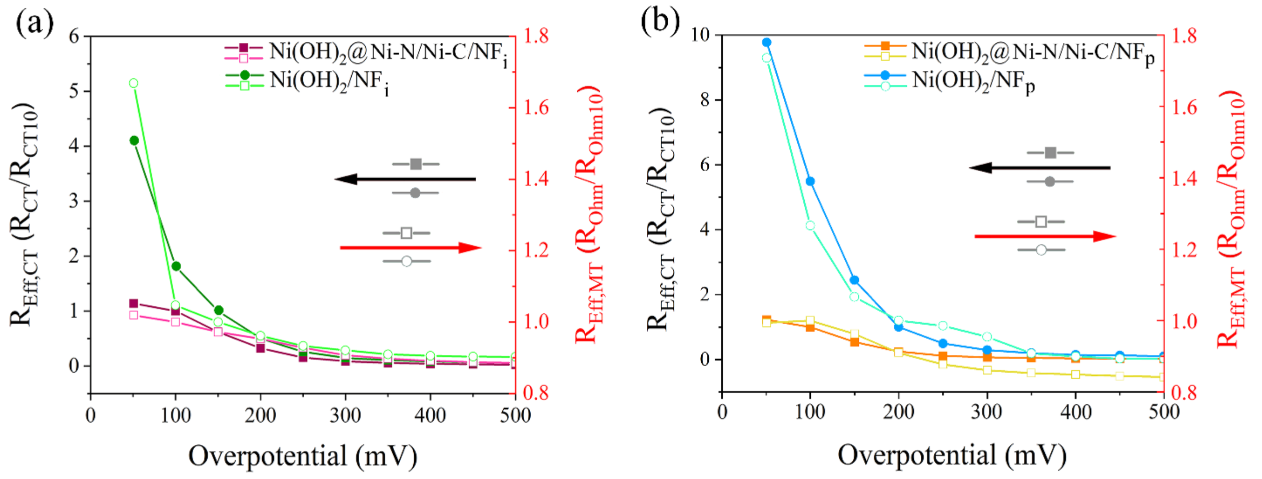

Figure S37. Effective interfacial charge transfer and mass transport of (a) $Ni(OH)_2$@Ni-N/Ni-C/NF_i and $Ni(OH)_2/NF_i$ and (b) $Ni(OH)_2$@Ni-N/Ni-C/NF_p and $Ni(OH)_2/NF_p$.

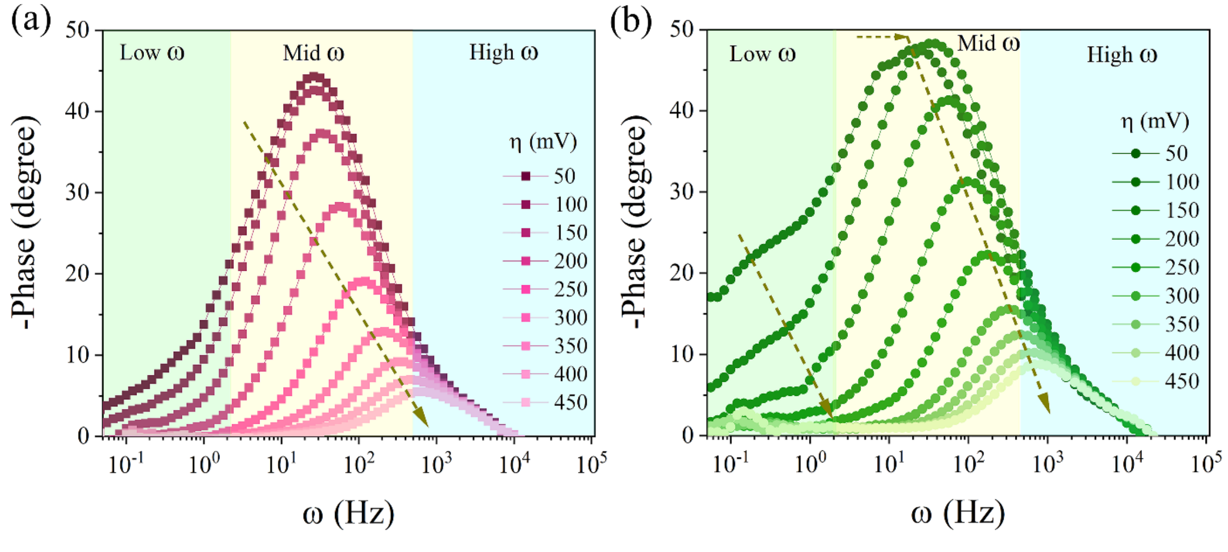

Figure S38. Bode-phase plots of (a) $Ni(OH)_2$@Ni-N/Ni-C/NF and (b) $Ni(OH)_2$/NF.

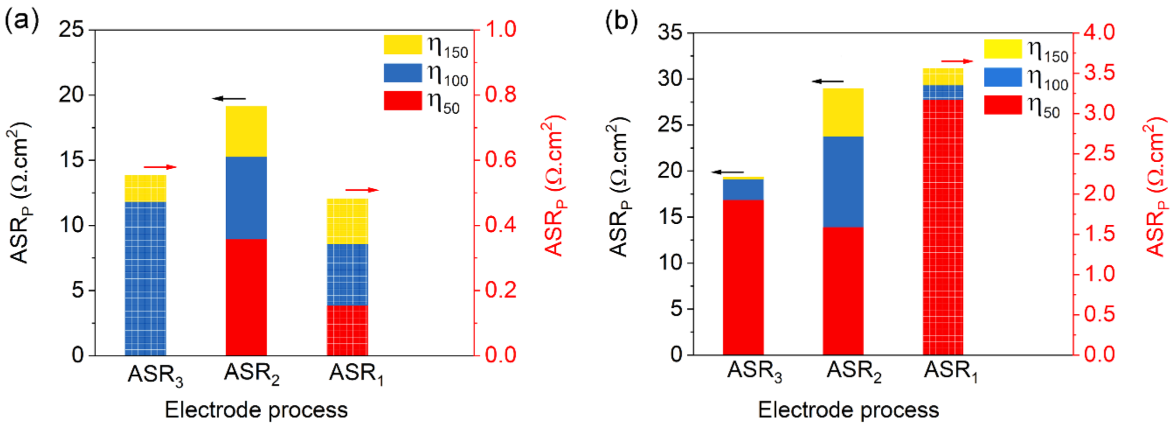

Figure S39. Potential-dependent ASR variation for (a) $Ni(OH)_2$@Ni-N/Ni-C/NF and (b) $Ni(OH)_2$/NF.

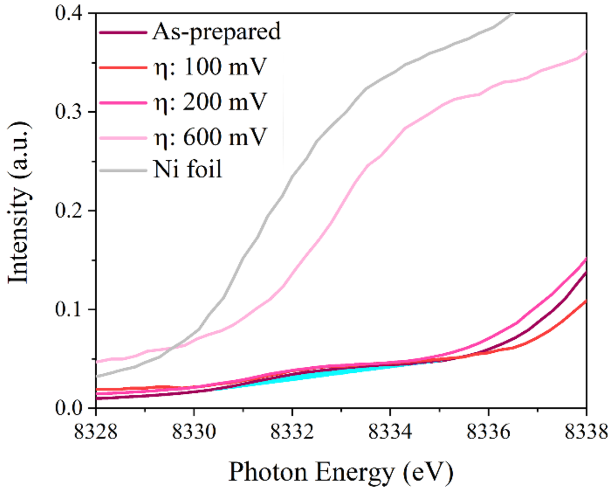

Figure S40. Comparison of the pre-edge for $Ni(OH)_2$@Ni-N/Ni-C/Ni/CFP with increasing HER overpotential.

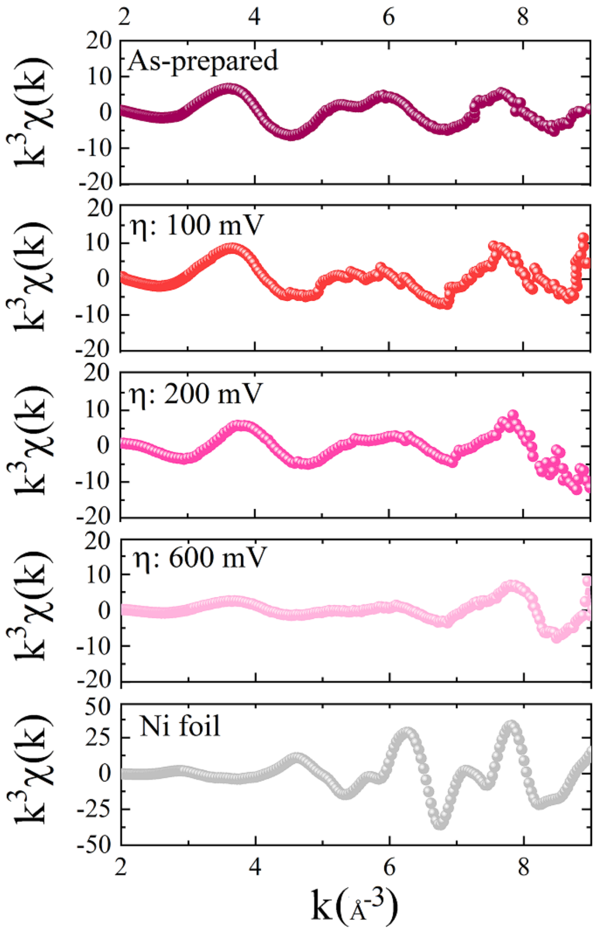

Figure S41. Quasi-operando measurements of Ni k edge EXAFS with increasing HER overpotential.

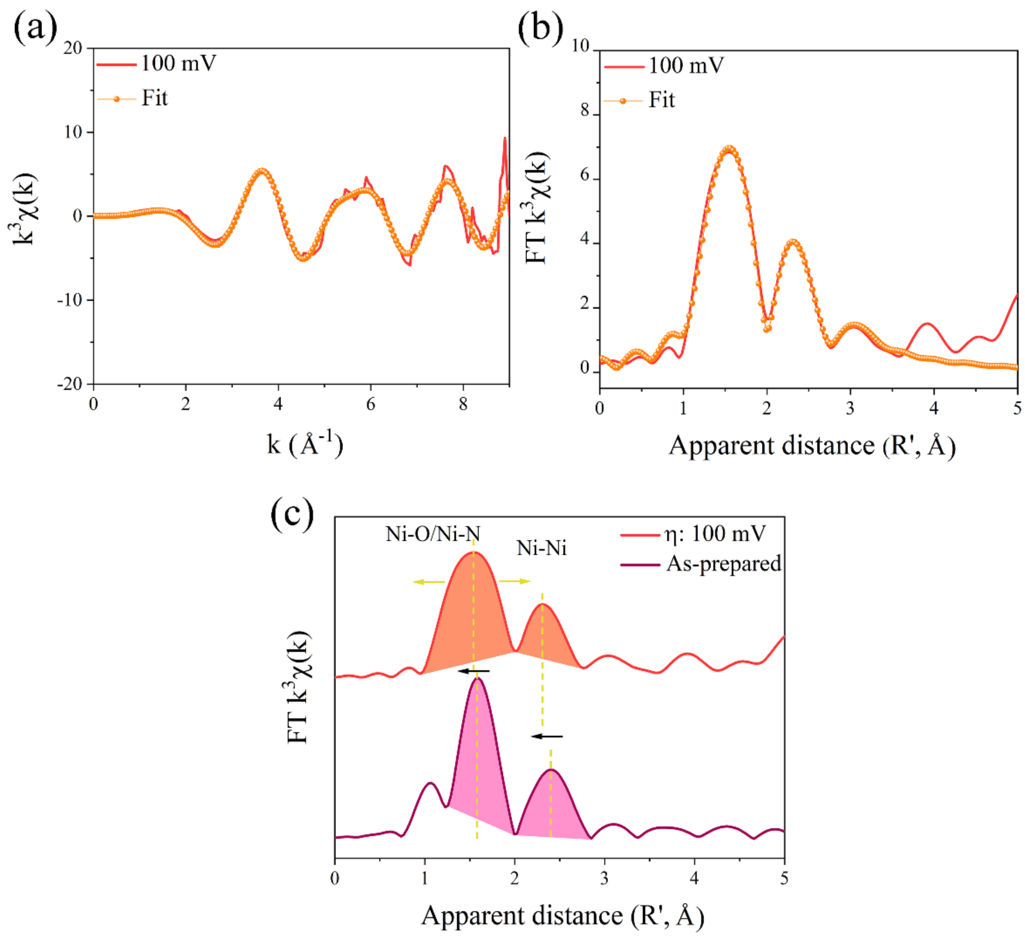

Figure S42. (a) and (b) EXAFS fitting of post-HER $Ni(OH)_2$@Ni-N/Ni-C/Ni/CFP at an overpotential of 100 mV. (c) Comparison of FT-EXAFS of as-prepared and treated $Ni(OH)_2$@Ni-N/Ni-C/Ni/CFP at 100 mV.

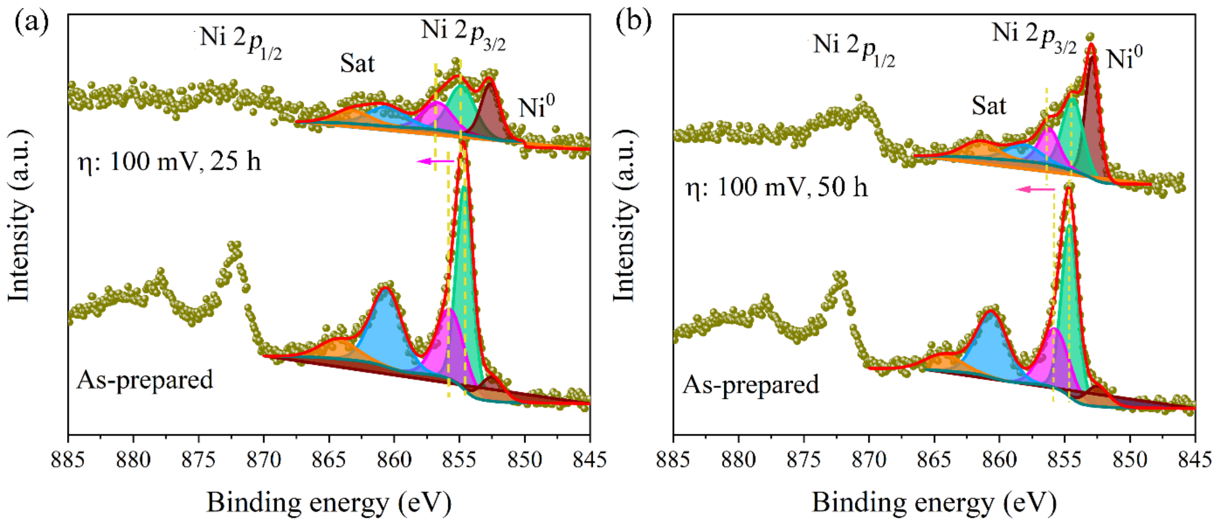

Figure S43. Comparison of Ni 2*p* core-level XPS profiles of as-prepared and treated $Ni(OH)_2$@Ni-N/Ni-C/NF at 100 mV after (a) 25 h and (b) 50 h chronopotentiometric tests.

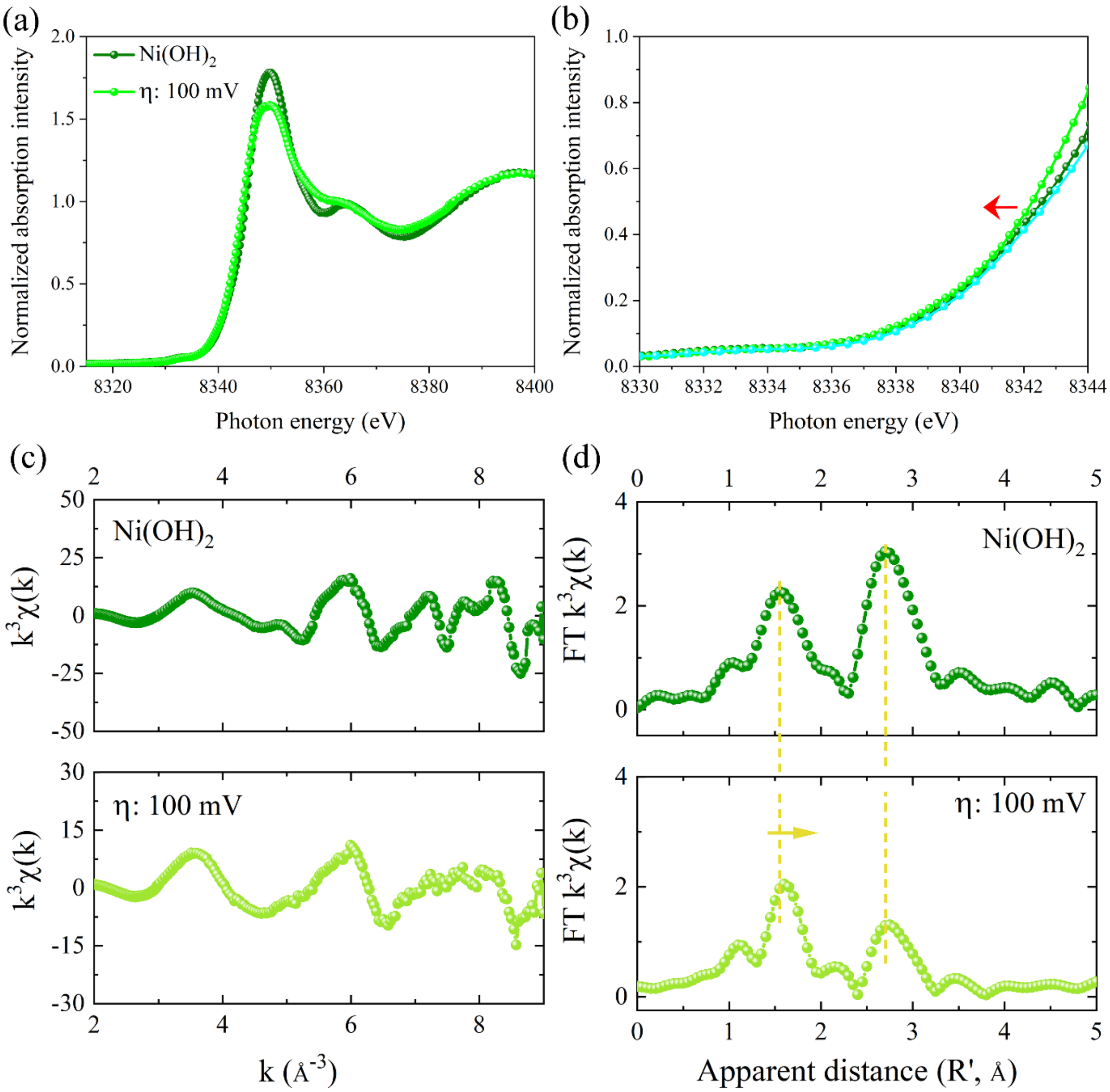

Figure S44. Comparison of (a) and (b) XANES spectra, (c) Ni k edge EXAFS, (d) Fourier transforms of EXAFS of as-prepared and post-HER $Ni(OH)_2$/Ni/CFP at an overpotential of 100 mV.

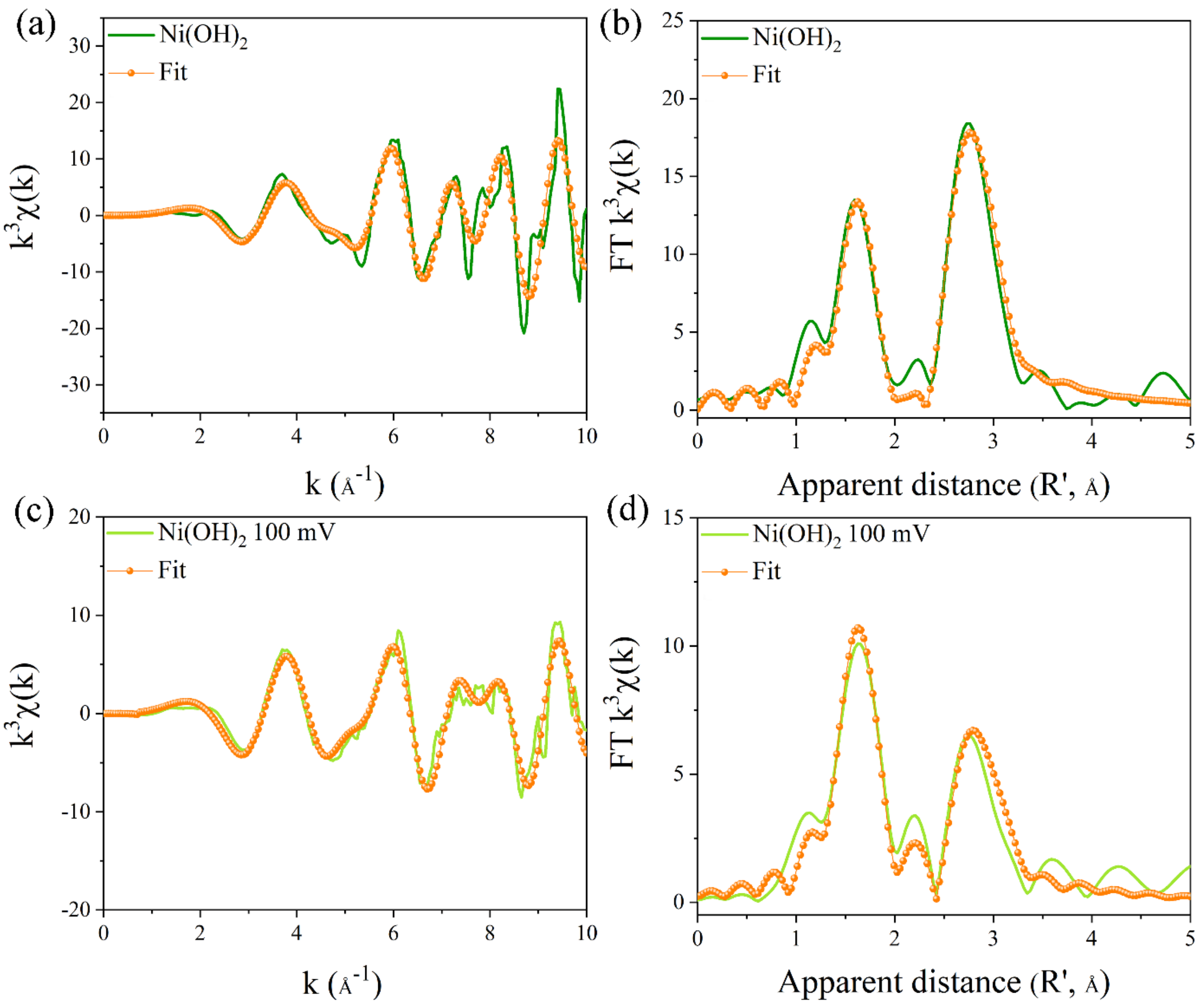

Figure S45. Comparison of EXAFS fitting of (a) and (b) as prepared, with (c) and (d) post-HER $Ni(OH)_2$/Ni/CFP.

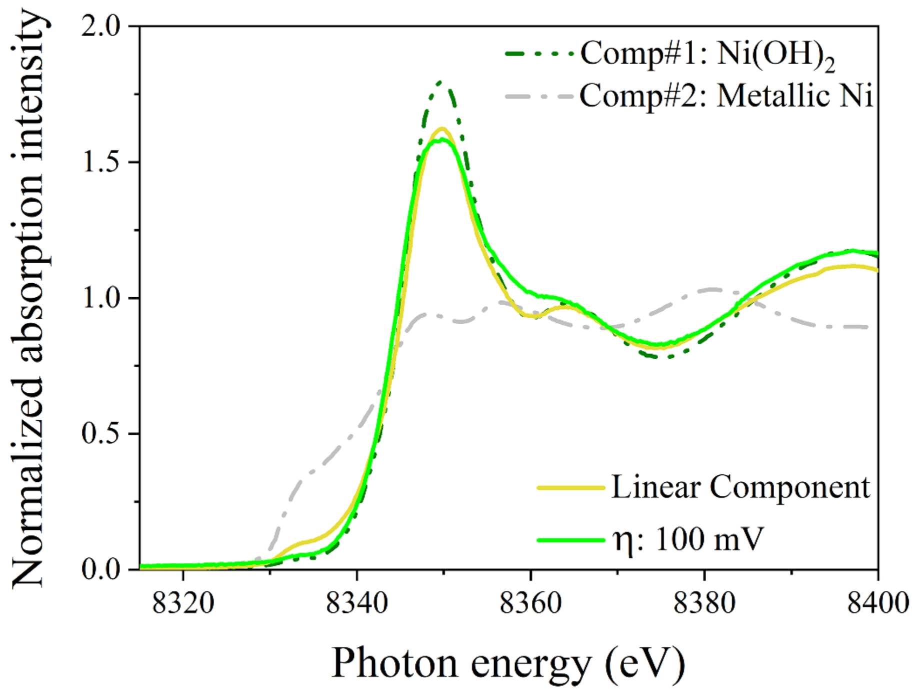

Figure S46. Comparison of LCA for post-HER $Ni(OH)_2$ XANES at 100 mV.

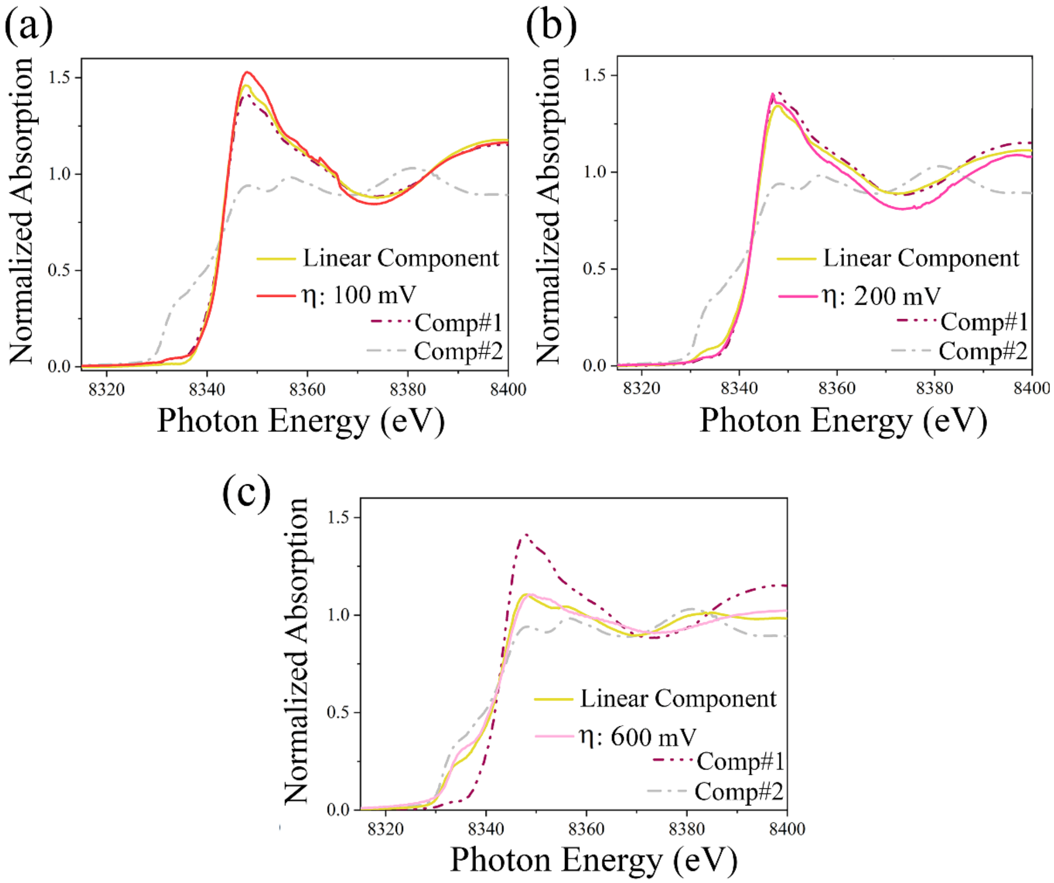

Figure S47. LCA graphs of $Ni(OH)_2$@Ni-N/Ni-C/Ni/CFP at (a) 100 mV, (b) 200 mV, and (c) 600 mV. Component 1: $Ni(OH)_2$@Ni-N/Ni-C and Component 2: Metallic Ni.

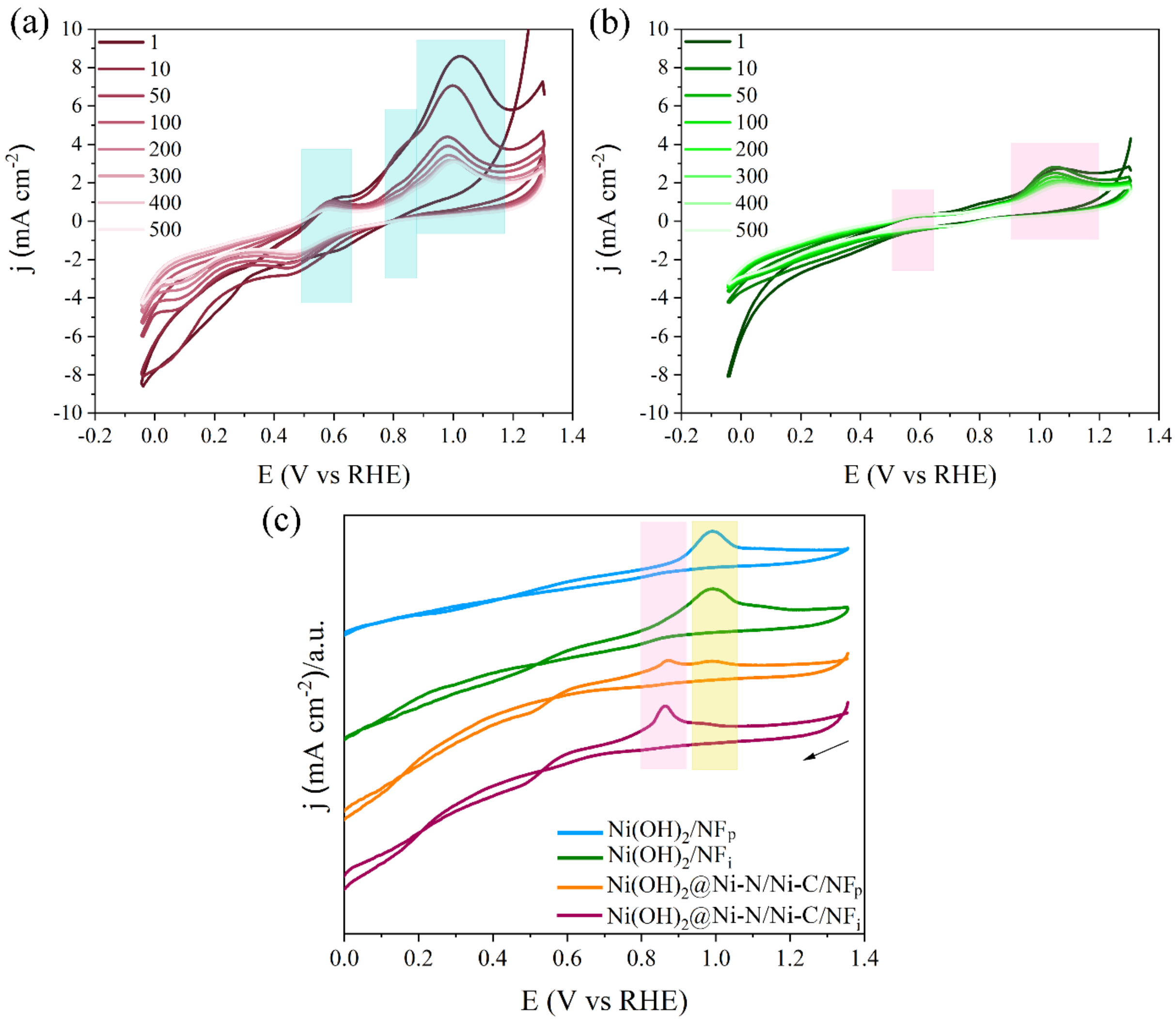

Figure S48. CV curves of as-prepared (a) $Ni(OH)_2$@Ni-N/Ni-C/NF and (b) $Ni(OH)_2$/NF for OH intermediate adsorption at 10 mV s^{-1} in 1 M KOH. (c) Comparison of CV curves obtained at 1 mV s^{-1} in 1 M KOH for the electrodes with added Ni ions ($Ni(OH)_2$@Ni-N/Ni-C/NF_i and $Ni(OH)_2$/NF_i) and with Ni from the pristine Ni foam ($Ni(OH)_2$@Ni-N/Ni-C/NF_p and $Ni(OH)_2$/NF_p) after HER polarization and obtaining of stable LSV curves.

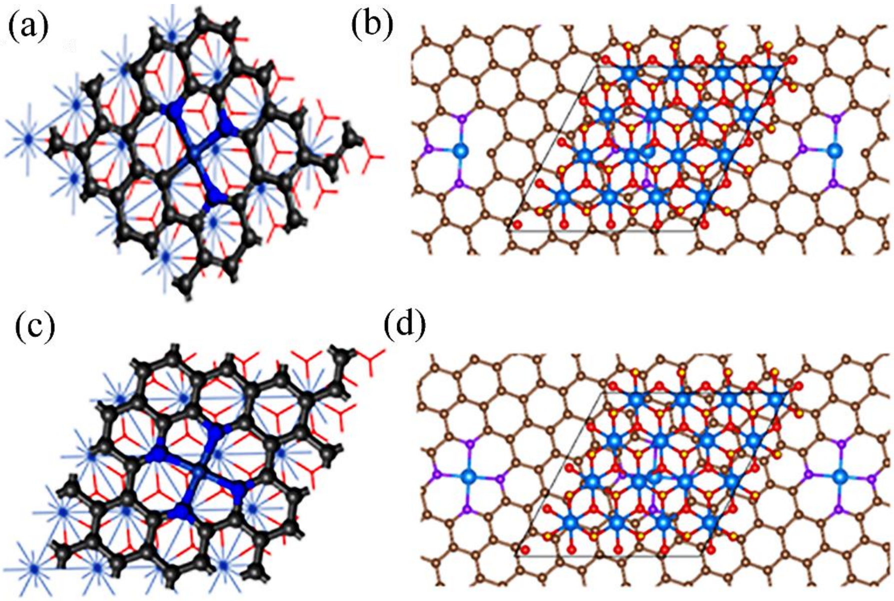

Figure S49. Atomic structure and in-plain slab models of (a) and (b) $Ni(OH)_2$@NiN_3-C, (c) and (d) $Ni(OH)_2$@NiN_4-C.

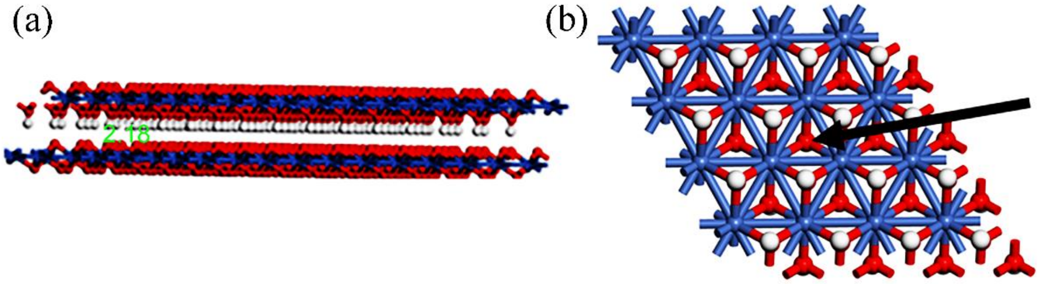

Figure S50. Atomic structure and in-plain slab models of monophasic $Ni(OH)_2$.

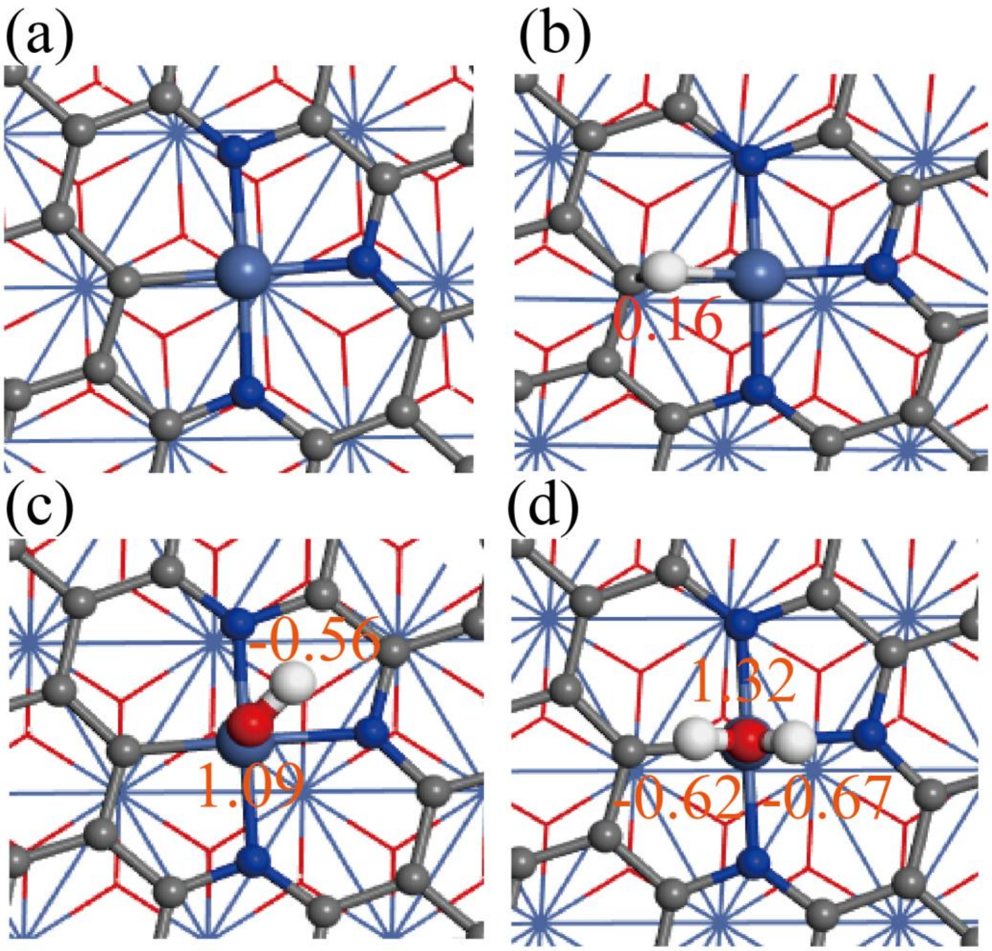

Figure S51. The loss and gain of Bader charges during the adsorption of (a) *H, (b) *OH, and (d) H_2O on the Ni active site in NiN_3-C phase in $Ni(OH)_2$@NiN_3-C.

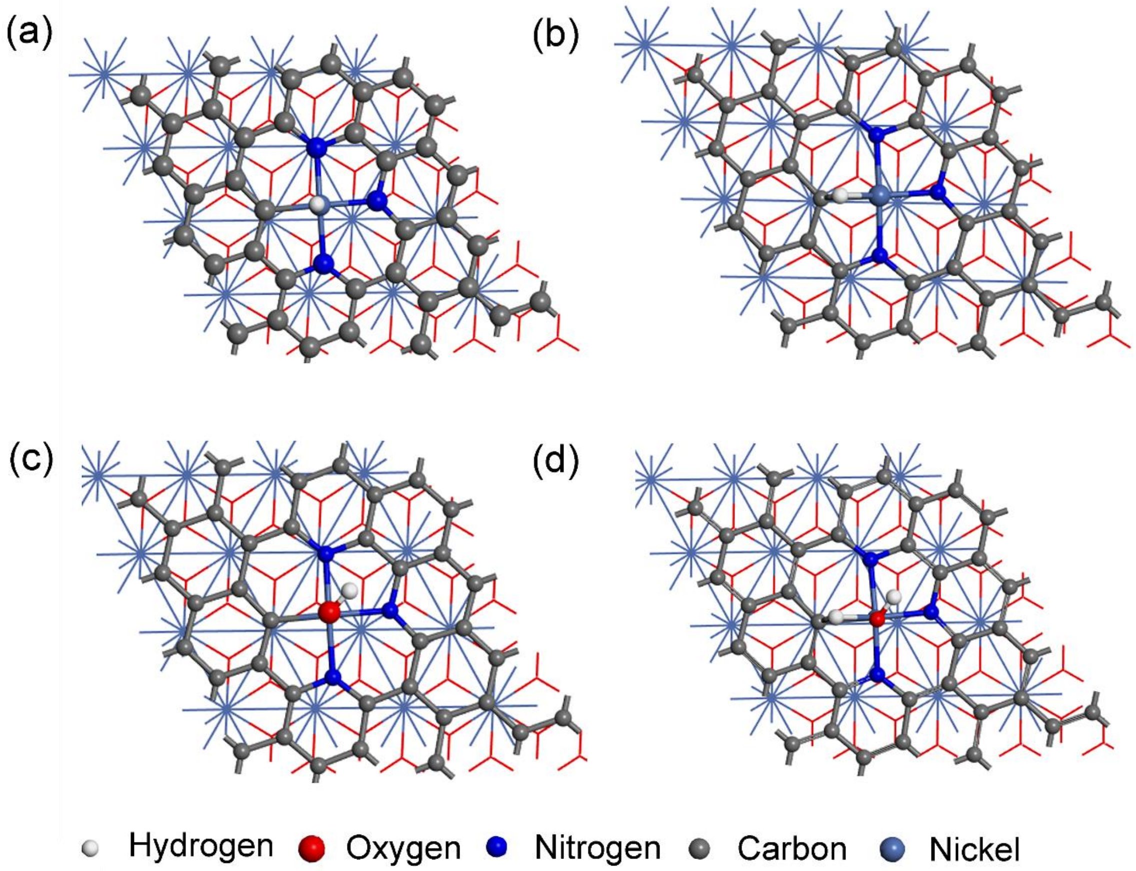

Figure S52. In-plain slab models of (a) *H adsorption on top Ni sites in $Ni(OH)_2$@NiN_3-C, (b) *H adsorption on bridge Ni sites in $Ni(OH)_2$@NiN_3-C, (c) *OH adsorption on top Ni sites in $Ni(OH)_2$@NiN_3-C, and (d) *H-*OH co-adsorption on $Ni(OH)_2$@NiN_3-C.

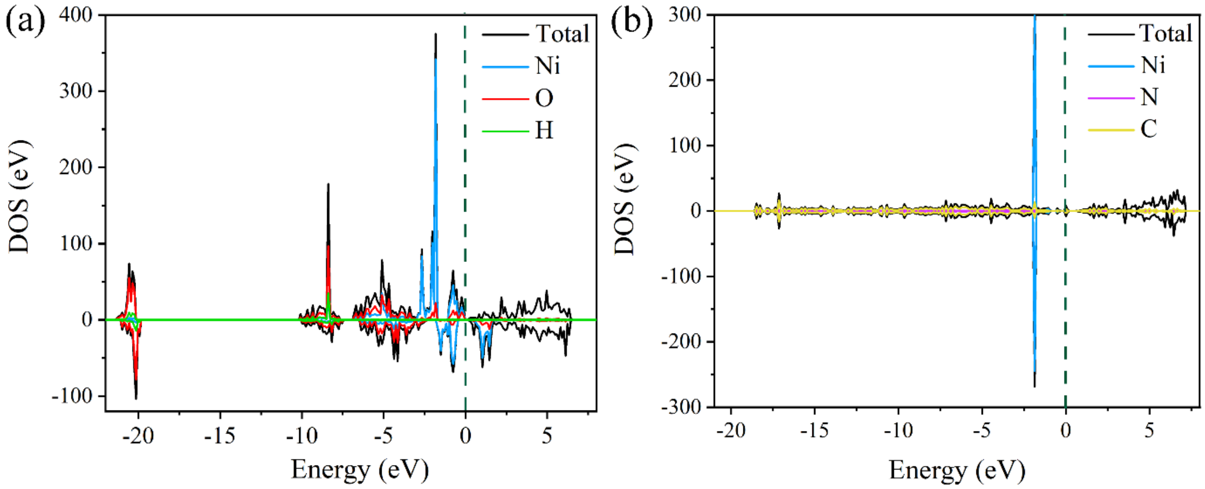

Figure S53. Calculated PDOS profiles of (a) $Ni(OH)_2$ and (b) NiN_3-C.

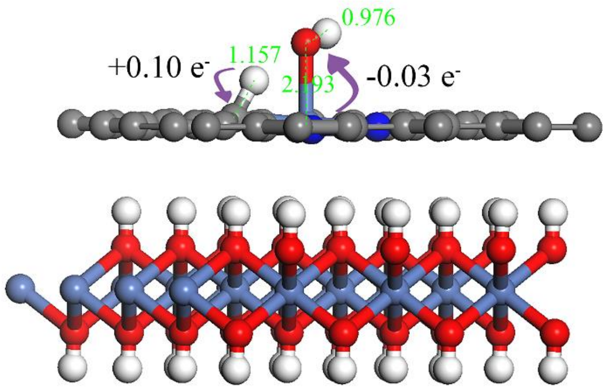

Figure S54. Bader charge variation in NiN_3-C phase for the co-adsorption of *H and *OH intermediates.

Table S1. Process parameters with their three observation levels.

Control parameter	Low	Medium	High
Concentration of ionic Ni (mM)	2.5	5	7.5
H_2O to EDA volume ratio (mL)	1/4	2/2	4/1
Reaction time (h)	3	4.5	6
Reaction temperature (°C)	120	150	180

Table S2. Normalized variables with their three normalized observation levels.

Normalised variable	Low	Medium	High
X_1	-1	0	1
X_2	-1	0	1
X_3	-1	0	1
X_4	-1	0	1

Table S3. Taguchi optimization orthogonal array (L_9) of four control parameters in solvothermal synthesis at three different levels.

Run	X_1	X_2	X_3	X_4
1	-1	-1	-1	-1
2	-1	0	0	0
3	-1	1	1	1
4	0	-1	0	1
5	0	0	1	-1
6	0	1	-1	0
7	1	-1	1	0
8	1	0	-1	1
9	1	1	0	-1

Table S4. Comparison of HER performance of $Ni(OH)_2$@Ni-N/Ni-C/NF with the reported Ni-based multiphasic and multimetallic electrocatalysts in KOH electrolyte.

Catalyst	Electrode	η10[a] (mV)	η100[b] (mV)	η1000[c] (mV)	S_j[d] (h)	b[e] (mV dec^{-1})	TOFj[f] (H_2 s^{-1})	Ref.
$Ni(OH)_2$@Ni-N/Ni-C	NF	68	141	223	$100_{500, 100}$	43.9	0.402_{100}	This work
Ni@$Ni(OH)_2$@NiOOH	CC[g]	45	-	-	200_{200}	99.6	-	[13]
Ni_3N@Ni	NF	66	-	-	75_{10}	38.0	-	[25]
Ni_3N@Ni	NF	12	64	-	50_{10}	29.3	-	[27]
Ni@Ni-N-C	EG[h]	147	-	-	10_{30}	114	-	[28]
$Ni(OH)_2$@Ni_3N	TM[i]	60	181	-	25_{15}	86.0	-	[19]
$NiCo_2S_4$@Ni_3S_2	NF	119	-	-	24_{30}	105.2	-	[29]
Ni_2P@$NiTe_2$	NF	62	143	-	50_{50}	80.0	-	[4]
Ni_3N@VN	NF	59	174	-	20_{10}	34.0	1.5[j]	[14]
Ni@CeF_3@VN	NF	33	110	-	241_{0}	37.1	0.08_{100}	[30]
NiO@Ni@Pt SA	CC	26	85	-	28_{20}	27.07	5.71_{50}	[31]
$Ni(OH)_2$@MoS_2	CC	80	-	-	16_{10}	60.0	-	[20]
Ni_3N@NiMoN	CC	31	200	-	20_{10}	64.0	0.265[k]	[18]
$Ni(OH)_2$@PtO_2 NS	TM	44	-	-	100_{20}	89.0	-	[22]
Ni_3Fe LDHs@Pt SA	GC[l]	45	-	-	12	54.3	-	[15]
Ni@WC	GC	77	-	-	24_{20}	68.6	-	[17]
NiS_2@MoS_2	GC	204	-	-	6_{10}	65.0	-	[32]

[a,b,c] Overpotentials at current densities of 10, 100, and 1000 mA cm^{-2}. [d] Stability at a given current density in mA cm^{-2}. [e] Tafel slope. [f] TOF values reported at a given overpotential in mV at alkaline HER conditions. [g] Carbon cloth. [h] Exfoliated graphene foil. [i] Titanium mesh. [j,k] The TOF values are reported at an overpotential of 100 mV. [l] Glassy carbon.

Table S5. Fitting parameters of the EXAFS spectrum of as-prepared $Ni(OH)_2$@Ni-N/Ni-C. Coordination number (CN), Debye-Waller factor (σ^2), interatomic distance (Reff). S02= 0.65, R-factor =0.044

Path	CN	σ^2	Reff
Ni-O/ Ni-N	6	0.0035	2.005
Ni-Ni	3	0.0032	2.846
Ni-Ni	2	0.0027	2.932

Table S6. Fitting parameters of the EXAFS spectrum of post-HER $Ni(OH)_2$@Ni-N/Ni-C at an overpotential of 100 mV. Coordination number (CN), Debye-Waller factor (σ^2), interatomic distance (Reff). S02= 1.178, R-factor =0.006

Path	CN	σ^2	Reff
Ni-O/ Ni-N	3	0.0019	2.04
Ni-O/ Ni-N	3	0.0020	2.19
Ni-Ni	2	0.0023	2.93
Ni-Ni	2	0.0040	3.10

References

1 Mora-Tamez, L. *et al.* Controlled Design of Phase- and Size-Tunable Monodisperse Ni2P Nanoparticles in a Phosphonium-Based Ionic Liquid through Response Surface Methodology. *Chemistry of Materials* **31**, 1552-1560, doi:10.1021/acs.chemmater.8b04518 (2019).

2 Sriram, P. *et al.* Hybridizing Strong Quadrupole Gap Plasmons Using Optimized Nanoantennas with Bilayer MoS_2 for Excellent Photo-Electrochemical Hydrogen Evolution. *Advanced Energy Materials* **8**, doi:10.1002/aenm.201801184 (2018).

3 Li, Y. *et al.* Ni-based 3D hierarchical heterostructures achieved by selective electrodeposition as a bifunctional electrocatalyst for overall water splitting. *Electrochimica Acta* **379**, doi:10.1016/j.electacta.2021.138042 (2021).

4 Li, Y. *et al.* Phosphine vapor-assisted construction of heterostructured Ni2P/NiTe2 catalysts for efficient hydrogen evolution. *Energy & Environmental Science* **13**, 1799-1807, doi:10.1039/d0ee00666a (2020).

5 Sun, H. *et al.* Bixbyite-type Ln2O3 as promoters of metallic Ni for alkaline electrocatalytic hydrogen evolution. *Nat Commun* **13**, 3857, doi:10.1038/s41467-022-31561-4 (2022).

6 Kresse, G. & Furthmuller, J. Efficiency of ab-initio total energy calculations for metals and semiconductors using a plane-wave basis set. *Comp Mater Sci* **6**, 15-50 (1996).

7 Kresse, G. & Furthmuller, J. Efficient iterative schemes for ab initio total-energy calculations using a plane-wave basis set. *Phys Rev B* **54**, 11169-11186 (1996).

8 Perdew, J. P., Burke, K. & Ernzerhof, M. Generalized gradient approximation made simple. *Phys Rev Lett* **77**, 3865-3868 (1996).

9 Blochl, P. E. Projector Augmented-Wave Method. *Phys Rev B* **50**, 17953-17979 (1994).

10 Monkhorst, H. J. & Pack, J. D. Special Points for Brillouin-Zone Integrations. *Phys Rev B* **13**, 5188-5192 (1976).

11 Methfessel, M. & Paxton, A. T. High-Precision Sampling for Brillouin-Zone Integration in Metals. *Phys Rev B* **40**, 3616-3621 (1989).

12 Klimes, J., Bowler, D. R. & Michaelides, A. Van der Waals density functionals applied to solids. *Phys Rev B* **83** (2011).

13 Rathore, D., Sharma, M. D., Sharma, A., Basu, M. & Pande, S. Aggregates of Ni/Ni(OH)2/NiOOH Nanoworms on Carbon Cloth for Electrocatalytic Hydrogen Evolution. *Langmuir* **36**, 14019-14030, doi:10.1021/acs.langmuir.0c02548 (2020).

14 Yan, H. *et al.* Anion-Modulated HER and OER Activities of 3D Ni-V-Based Interstitial Compound Heterojunctions for High-Efficiency and Stable Overall Water Splitting. *Adv Mater* **31**, e1901174, doi:10.1002/adma.201901174 (2019).

15 Chen, W. *et al.* Deciphering the alternating synergy between interlayer Pt single-atom and NiFe layered double hydroxide for overall water splitting. *Energy & Environmental Science* **14**, 6428-6440, doi:10.1039/d1ee01395e (2021).

16 Xin, Y., Kan, X., Gan, L. Y. & Zhang, Z. Heterogeneous Bimetallic Phosphide/Sulfide Nanocomposite for Efficient Solar-Energy-Driven Overall Water Splitting. *Acs Nano* **11**, 10303-10312, doi:10.1021/acsnano.7b05020 (2017).

17 Ma, Y.-Y. *et al.* Highly efficient hydrogen evolution triggered by a multi-interfacial Ni/WC hybrid electrocatalyst. *Energy & Environmental Science* **11**, 2114-2123, doi:10.1039/c8ee01129j (2018).

18 Wu, A. *et al.* Integrating the active OER and HER components as the heterostructures for the efficient overall water splitting. *Nano Energy* **44**, 353-363, doi:10.1016/j.nanoen.2017.11.045 (2018).

19 Gao, M., Chen, L., Zhang, Z., Sun, X. & Zhang, S. Interface engineering of the Ni(OH)2–Ni3N nanoarray heterostructure for the alkaline hydrogen evolution reaction. *Journal of Materials Chemistry A* **6**, 833-836, doi:10.1039/c7ta08907d (2018).

20 Zhang, B. *et al.* Interface engineering: The Ni(OH) 2 /MoS 2 heterostructure for highly efficient alkaline hydrogen evolution. *Nano Energy* **37**, 74-80, doi:10.1016/j.nanoen.2017.05.011 (2017).

21 Yin, J. *et al.* Ni-C-N Nanosheets as Catalyst for Hydrogen Evolution Reaction. *J Am Chem Soc* **138**, 14546-14549, doi:10.1021/jacs.6b09351 (2016).

22 Xie, L. *et al.* A Ni(OH)2–PtO2 hybrid nanosheet array with ultralow Pt loading toward efficient and durable alkaline hydrogen evolution. *Journal of Materials Chemistry A* **6**, 1967-1970, doi:10.1039/c7ta09990h (2018).

23 Li, H. *et al.* Systematic design of superaerophobic nanotube-array electrode comprised of transition-metal sulfides for overall water splitting. *Nat Commun* **9**, 2452, doi:10.1038/s41467-018-04888-0 (2018).

24 Kim, J. *et al.* Tailoring Binding Abilities by Incorporating Oxophilic Transition Metals on 3D Nanostructured Ni Arrays for Accelerated Alkaline Hydrogen Evolution Reaction. *J Am Chem Soc* **143**, 1399-1408, doi:10.1021/jacs.0c10661 (2021).

25 Zhang, D. *et al.* Unconventional direct synthesis of Ni3N/Ni with N-vacancies for efficient and stable hydrogen evolution. *Energy & Environmental Science* **15**, 185-195, doi:10.1039/d1ee02013g (2022).

26 Liu, B. *et al.* Unconventional Nickel Nitride Enriched with Nitrogen Vacancies as a High-Efficiency Electrocatalyst for Hydrogen Evolution. *Adv Sci (Weinh)* **5**, 1800406, doi:10.1002/advs.201800406 (2018).

27 Song, F. *et al.* Interfacing nickel nitride and nickel boosts both electrocatalytic hydrogen evolution and oxidation reactions. *Nat Commun* **9**, 4531, doi:10.1038/s41467-018-06728-7 (2018).

28 Lei, C. *et al.* Efficient alkaline hydrogen evolution on atomically dispersed Ni–Nx Species anchored porous carbon with embedded Ni nanoparticles by accelerating water dissociation kinetics. *Energy & Environmental Science* **12**, 149-156, doi:10.1039/c8ee01841c (2019).

29 Liu, H. *et al.* Heteromorphic NiCo2S4/Ni3S2/Ni Foam as a Self-Standing Electrode for Hydrogen Evolution Reaction in Alkaline Solution. *ACS Appl Mater Interfaces* **10**, 10890-10897, doi:10.1021/acsami.8b00296 (2018).

30 Zhou, P. *et al.* Construction of Nickel-Based Dual Heterointerfaces towards Accelerated Alkaline Hydrogen Evolution via Boosting Multi-Step Elementary Reaction. *Advanced Functional Materials* **31**, doi:10.1002/adfm.202104827 (2021).

31 Zhou, K. L. *et al.* Platinum single-atom catalyst coupled with transition metal/metal oxide heterostructure for accelerating alkaline hydrogen evolution reaction. *Nat Commun* **12**, 3783, doi:10.1038/s41467-021-24079-8 (2021).

32 Kuang, P., Tong, T., Fan, K. & Yu, J. In Situ Fabrication of Ni–Mo Bimetal Sulfide Hybrid as an Efficient Electrocatalyst for Hydrogen Evolution over a Wide pH Range. *ACS Catalysis* **7**, 6179-6187, doi:10.1021/acscatal.7b02225 (2017).
